# A first-principles-based high fidelity, high throughput approach for the design of high entropy alloys

**DOI:** 10.1038/s41598-022-16082-w

**Published:** 2022-07-13

**Authors:** V. Sorkin, Z. G. Yu, S. Chen, Teck L. Tan, Z. H. Aitken, Y. W. Zhang

**Affiliations:** Institute of High Performance Computing, A*STAR, Singapore, 138632 Singapore

**Keywords:** Materials science, Theory and computation, Computational methods

## Abstract

Here, we present a preselected small set of ordered structures (PSSOS) method, a first principles-based high fidelity (HF), high throughput (HT) approach, for fast screening of the large composition space of high entropy alloys (HEAs) to select the most energetically stable, single-phase HEAs. Taking quinary AlCoCrFeNi HEA as an example system, we performed PSSOS calculations on the formation energies and mass densities of 8801 compositions in both FCC and BCC lattices and selected five most stable FCC and BCC HEAs for detailed analysis. The calculation results from the PSSOS approach were compared with existing experimental and first-principles data, and the good agreement was achieved. We also compared the PSSOS with the special quasi-random structures (SQS) method, and found that with a comparable accuracy, the PSSOS significantly outperforms the SQS in efficiency, making it ideal for HF, HT calculations of HEAs.

## Introduction

High entropy alloys (HEAs)^1–4^, which contain multiple principal elements, have recently attracted considerable research interest due to their exceptional mechanical and physical properties, such as high corrosion, wear, fatigue resistances, yield strength, ductility, and thermal stability^1,2^, often outperforming traditional alloys and super-alloys^2^, especially at low and high temperatures^3,4^. However, the design (compositional and chemical) space of HEAs is vast, which makes the use of the conventional methods based on lengthy, costly, high-fidelity (HF) approaches, a daunting task. An efficient strategy is thus highly demanded to screen and design HEAs with optimal mechanical and physical properties for specific applications^5,6^.

Machine learning (ML) is emerging as a powerful tool for inductive screening and phase selection of HEAs^7–11^. However, the accuracy of ML is strongly dependent on the size and quality of used dataset ^12^. The lack of a large-size, high-quality dataset for HEAs is currently a major challenge for using ML. Due to the significant challenges in developing high throughput (HT) experimental techniques to produce large-size, high-quality dataset^13^, HT computations present a promising alternative solution.

Several computational methods have been employed to study HEAs, such as first-principles^14^, CALPHAD^15,16^, molecular dynamics (MD)^17–19^. Yet these methods suffer from the serious drawbacks either due to their low efficiency or low accuracy, and thus are not suitable for producing a large-size, high-quality dataset of HEAs. For example, MD and CALPHAD are semi-empirical methods, and there is significant uncertainty in their accuracy. To our knowledge, four major first-principles-based methods have been developed to study HEAs^20–29^. The first one is based on coherent potential approximation (CPA)^20–22,30^, the accuracy of which is limited by its mean-field nature (neglecting all the local environmentally-dependent effects)^31^. The second is the virtual crystal approximation (VCA), which is another commonly used method to study HEAs in solid solution state^27–29^. Though HEAs do not have translational symmetry, the VCA recovers it by replacing the potential with a periodic, composite one, which is constructed by averaging the constituent atoms^28,32^. Theoretically, the VCA approach is of great versatility, but, like CPA, the VCA does not describe different local atomic environments, in particular, lattice distortion in HEAs. The third method is based on the special quasi random structures (SQS)^23–25^ containing a few hundreds of atoms. Although the SQS method is accurate, its application to HEAs is hindered by its high computational cost, which scales as ~ $$O(N$$^3^$$)$$ with the number of atoms. The last method is based on the small set of ordered structures (SSOS) containing several atoms^20,26^, which is currently considered the most promising approach for HF, HT computations of HEAs.

In the SSOS method, the properties of an HEA are calculated as a weighted average over a selected set of small ordered structures (SOS)^20,26^. Since the properties of SOS are calculated using density functional theory (DFT), it is considered a first-principles-based method. It was initially proposed to deal with equimolar HEAs^26^, and only a single set of SOS was found. Subsequently, a set of SSOS solutions were found, and an averaging scheme was proposed to achieve more accurate, more robust solutions^33^. Recently, this method was also extended to deal with HEAs with non-equimolar compositions^34^ and short range order^35^. Although significant progress has been made in developing the SSOS method, a couple of challenges still stand in its way for general use in HF, HT calculations of HEAs. First, in the full design space of HEAs, the number of SOS structures required to construct the SSOS solutions is vast, causing the difficulty in selecting SOS structures. Second, the DFT calculations for many SOSs are too expensive, making the SSOS method lose its computational efficiency. These challenges hinder the application of the SSOS method for constructing a large-size, high-quality dataset.

By addressing the challenges in the SSOS method, in this work, we propose a preselected SSOS (PSSOS) approach that enables HF, HT screening and design of single-phase HEAs. Using experimentally well-studied quinary AlCoCrFeNi^36–38^ as an example, we employ PSSOS to calculate the formation energy, mass density, and lattice constant in the large composition HEA space, and identify the five most energetically stable compositions with both the BCC and FCC lattices for detailed analysis. Our PSSOS calculation results are compared with the existing experimental data, and a good agreement is found. Our PSSOS approach is also contrasted with the SQS method. It shows that the accuracy of our PSSOS approach is comparable with the SQS method. Since the compatibility of non-equimolar compositions often requires large SQS samples containing at least a thousand atoms, the PSSOS approach significantly outperforms the SQS method in terms of efficiency, making HF, HT screening and design of HEAs a reality.

## Results

### Outline of the PSSOS approach

**Figure 1:**
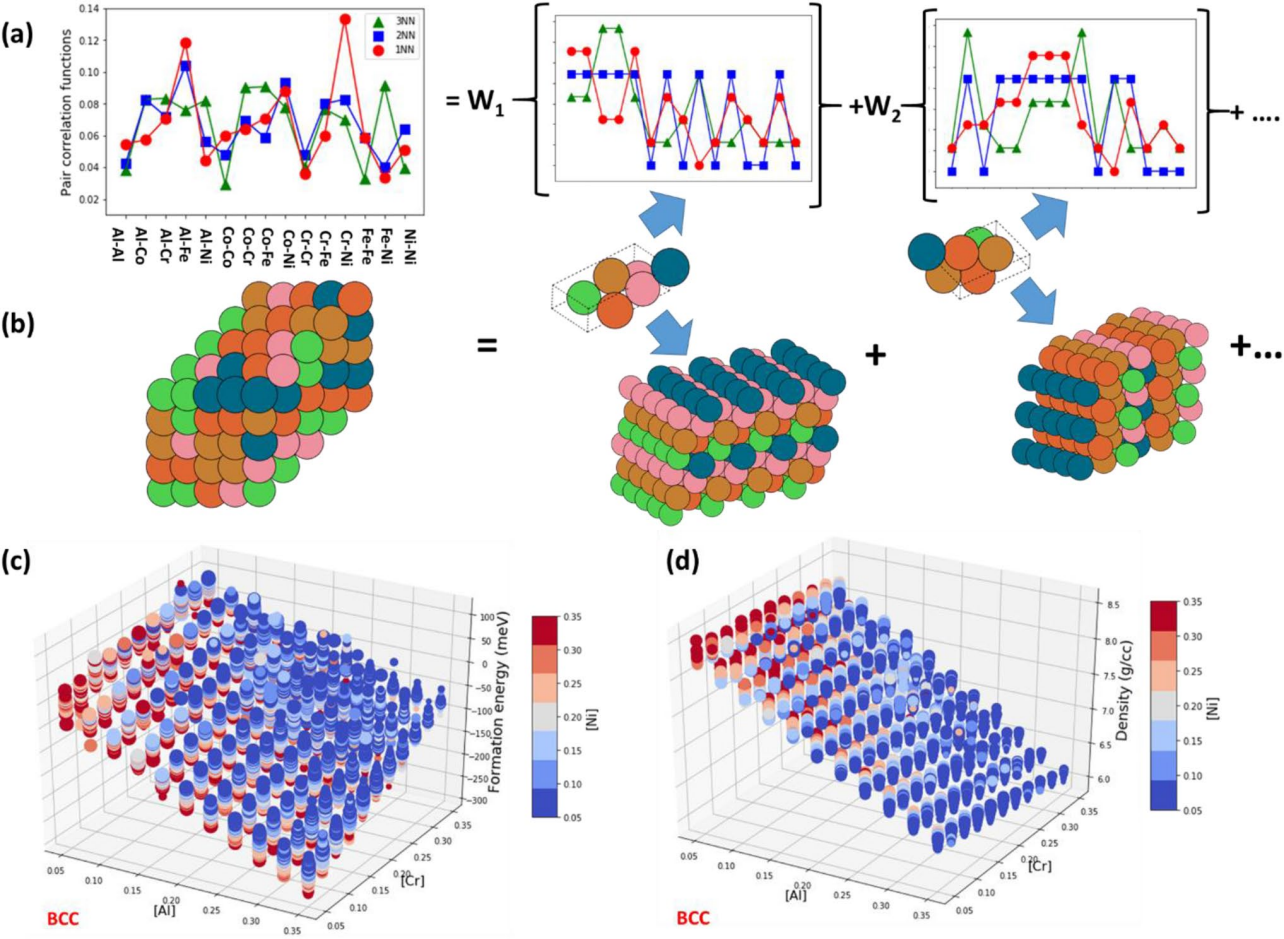
(**a**), (**b**) Schematic representation of the PSSOS method: (**a**) For a given composition of a quinary HEA in the ideal solid solution, one calculates the pair correlation functions, which must be precisely matched with a linear combination of the weighted pair correlation functions of a set of selected SOS. In our calculations, the pair correlation functions are matched up to 3rd nearest neighbor range with that of the ideal solid solution. The target SQS HEA sample and two 6-atom SOS samples taken from the preselected SOS set with their pair correlation functions are illustrated in (**b**). For each SOS 6-atom sample, its supercell version, obtained by its replication along the three directions, is presented to illustrate its ordered structure. (**c**), (**d**) The formation energy per atom (**c**) and mass density (**d**) of AlCoCrFeNi HEAs in BCC lattice structure as a function of the molar fraction of Al and Cr. Marker color indicates the molar fraction of Ni, while marker size corresponds to the molar fraction of Co for a given HEA composition.

More details about the PSSOS method can be found in the ‘Method’ section. The central idea of the SSOS method^20,26,31,33^ is to use a set of SOS to model an HEA with a given composition. Symmetry-unique SOS are constructed by using non-conventional, non-primitive unit cells with cubic lattices. Examples of these SOS containing a small number of atoms per unit cell are shown in Fig. 1. Each SOS is characterized by its own pair correlation functions, which describe the atomistic neighborhood of every constituent element and serve as its unique identification (‘fingerprints’). In principle, the complete set of all feasible SOS should be constructed and then optimized using DFT. Then, a small subset of SOS is selected from the complete set by matching the pair-correlation functions of a given HEA composition by a linear combination of pair correlation functions of the chosen SOS subset. For an ideal solid solution phase of HEA, its corresponding pair-correlation functions can be calculated analytically^35^. In our calculations, the HEA pair correlation functions are precisely matched up to the 3rd nearest neighbor (NN) range (see Fig. 1a). The properties of a selected HEA are calculated as a weighted average over those of the chosen SOS, which constitutes an SSOS solution. For example, the HEA energy $E$ can be calculated as: $E = \sum\nolimits_{i = 1}^{n} {w_{i} E_{i} ,}$ where $w_{i}$ is the weight of the $i{\text{th}}$ SOS, $E_{i}$ is the energy of the $i{\text{th}}$ SOS, and *n* is the number of SOS per an SSOS solution^26^.

To screen the entire HEA space, it is impractical to use the SSOS method as it requires construction and optimization of the complete set of all feasible SOS by DFT. To overcome this difficulty, we first choose SOS with only 5, 6, 7 atoms in the present work. Even with such choices, the total number of possible SOS is still more than 50,000 (see ‘Method’). To further reduce the number of SOS required, we adopt a new approach: first, the most frequent SOS structures (N ~ 1,500) are identified by screening the entire HEA space, and then their geometries are optimized, and their properties are calculated with DFT. We then look for SSOS solutions by taking SOS only from this small preselected SOS subset. We demonstrate (see ‘Method’ for details) that with comparable accuracy, the PSSOS approach is much faster than the SQS method, making it feasible for HF, HT calculations of HEAs.

### Systematic exploration of the composition space

**Figure 2:**
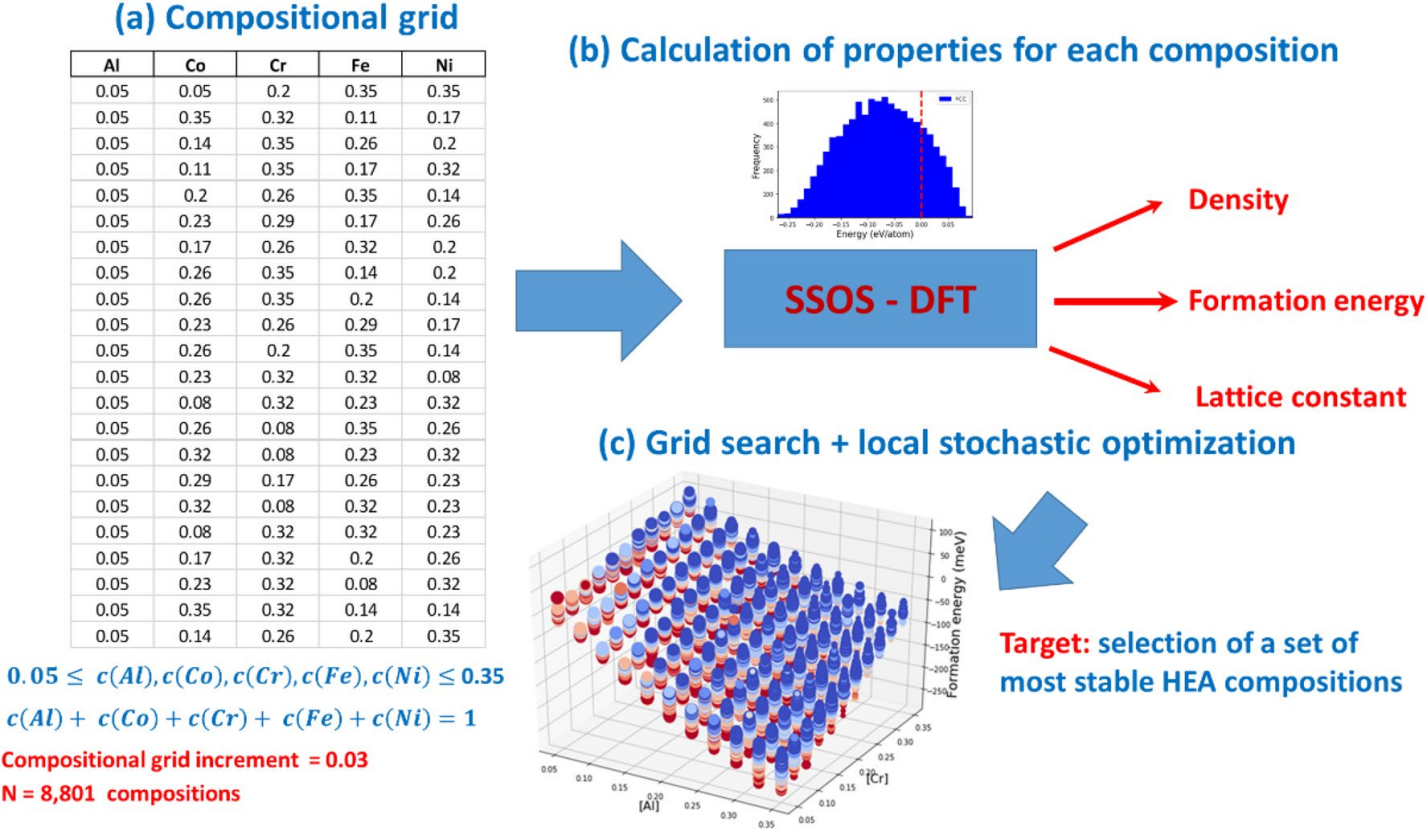
Schematic showing the compositional screening of a quinary HEA (AlCoCrFeNi) via the PSSOS method: (**a**) Construction of a compositional grid with a step size of 3% using *N* = 8801 compositions. Constraints on the molar fraction of constituent elements are indicated. (**b**) Calculations of the formation energy per atom, mass density and lattice constant for the constructed compositions of AlCoCrFeNi HEA with BCC and FCC lattices by the PSSOS method. (**c**) Selection of the most stable HEA compositions (with the lowest formation energies) for AlCoCrFeNi HEA, considering both BCC and FCC lattices.

The composition space of the AlCoCrFeNi HEA with FCC and BCC lattices, represented by a grid, was systematically explored by the PSSOS method. We set the lower (5%) and upper (35%) limits for the molar fractions of each constituent element and selected the molar fraction increment as: $\Delta =$ 3%. In total, 8,801 HEA compositions were constructed (see Fig. 2), and the formation energies and densities were calculated. Only the top-five most energetically stable compositions were selected for a second optimization step. Using the stochastic hill climbing method^39^, we examined the off-grid neighborhood of the top five compositions and found several compositions with an even lower formation energy for both the FCC and BCC lattices (see Table 1 and Supplementary Table S2).

**Table 1:** The top-five most energetically stable compositions for the AlCoCrFeNi HEA with a BCC lattice.

AlCoCrFeNi composition	Formation energy (eV/atom)		Density (g/cm^3^)		Young’s modulus (GPa)	
	BCC	FCC	BCC	FCC	BCC	FCC
[0.35,0.22,0.05,0.05,0.33]	− 0.281 ± 0.006	− 0.263 ± 0.004	6.64 ± 0.01	6.60 ± 0.01	216 ± 5	167 ± 5
[0.35,0.17,0.05,0.08,0.35]	− 0.280 ± 0.005	− 0.264 ± 0.003	6.61 ± 0.01	6.57 ± 0.01	205 ± 5	161 ± 4
[0.35,0.23,0.05,0.05,0.32]	− 0.279 ± 0.005	− 0.261 ± 0.004	6.64 ± 0.01	6.61 ± 0.01	216 ± 4	169 ± 3
[0.35,0.15,0.05,0.11,0.34]	− 0.275 ± 0.006	− 0.258 ± 0.004	6.58 ± 0.01	6.53 ± 0.01	207 ± 3	157 ± 4
[0.35,0.16,0.06,0.11,0.32]	− 0.265 ± 0.005	− 0.250 ± 0.004	6.56 ± 0.01	6.52 ± 0.01	208 ± 4	159 ± 3

The calculated formation energy per atom, mass density and Young’s modulus are compared with those with an FCC lattice at the same HEA composition. Error bars indicate the standard deviation of averaging over a set of SSOS solutions.

### Formation energy

The formation energy (per atom) of the AlCoCrFeNi HEA with a BCC lattice is plotted against the molar fraction of Al and Cr in a three-dimensional plot as shown in Fig. 2c (see also Supplementary Fig. S1 for FCC lattice). Since the composition space of the AlCoCrFeNi HEA is described by the four independent molar fractions, we indicate the molar fraction of Ni by marker color and the molar fraction of Co by marker size. As can be seen in Fig. 2c, the formation energy manifold is represented by an inclined plane.

**Figure 3:**
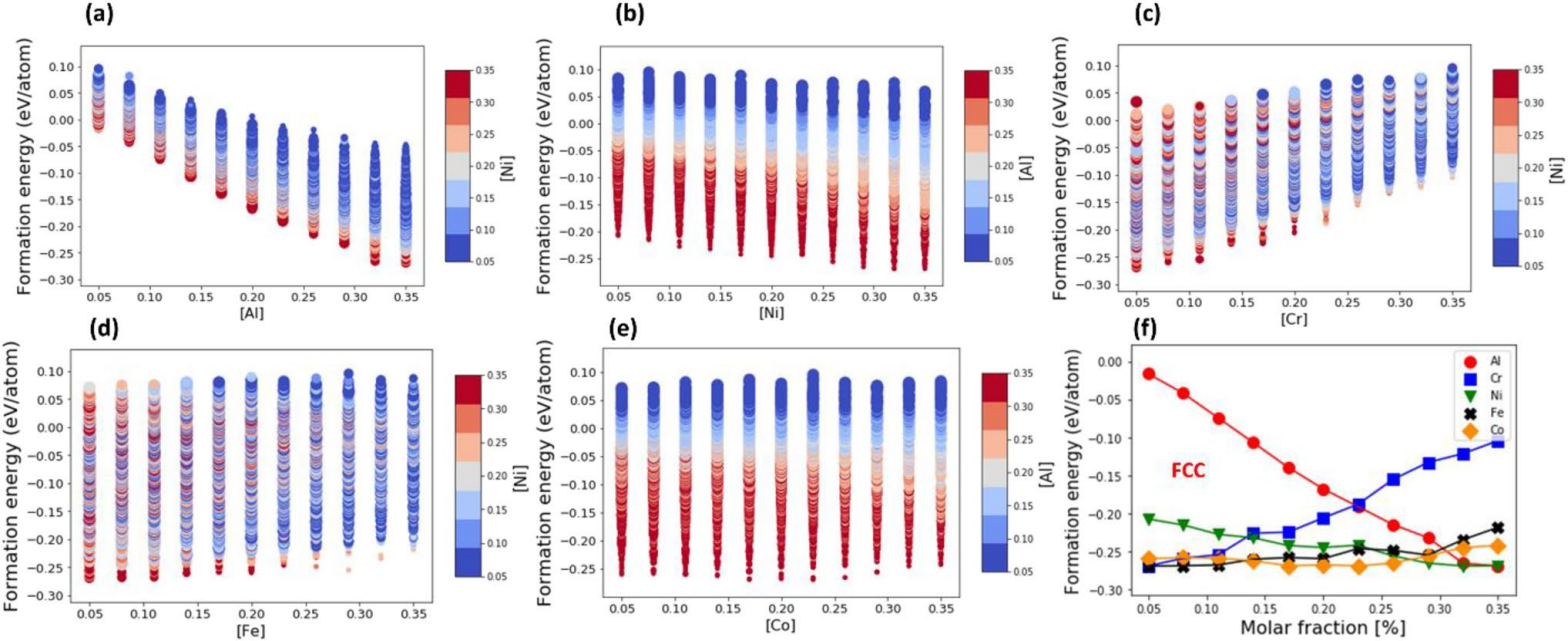
The effect of molar fraction of constituent elements on the formation energy of FCC AlCoCrFeNi. The formation energy of the AlCoCrFeNi HEA is plotted vs. molar fraction of Al (**a**), Ni (**b**), Cr (**c**), Fe (**d**) and Co (**e**). For a given HEA composition, marker color indicates the molar fraction of Ni in (**a**), (**f**), (**d**) and Al in (**b**), (**e**). Marker size corresponds to the molar fraction of Co in (**a**), (**f**), (**d**) and Cr in (**b**), (**e**). (**f**) The formation energy minimum of the AlCoCrFeNi HEA with an FCC lattice as a function of molar fraction of constituent elements: Al (red circles), Cr (blue squares), Ni (green triangles), Fe (black crosses) and Co (orange diamonds).

Figure 3 shows the formation energy *vs* the molar fraction of the constituent elements for the AlCoCrFeNi HEA with an FCC lattice (see also Supplementary Fig. S2 for BCC lattice). The formation energies of HEA compositions with equal molar fraction of one of their constituent elements are represented by a stacked column. Size and color of markers indicate the molar fractions of two other principal elements. The bottom of each column corresponds to the formation energy minimum at a given molar fraction (see Fig. 3f).

As illustrated in Fig. 3, the formation energy of AlCoCrFeNi with an FCC lattice is highly dependent on the molar fraction of specific constituent elements. For example, an increase in the molar fraction of Al results in a nearly linear reduction in the formation energy as shown in Fig. 3a (see also the red line with circles in Fig. 3f). Marker color indicates that an increase in the molar fraction of Ni also lowers the formation energy (see Fig. 3b, and the green curve with triangles in Fig. 3f). Yet, the effect of Ni is significantly weaker than that of Al: the mean slope for Ni in Fig. 3f is smaller than that of Al.

An increase in the molar fraction of Cr, in contrast to Al and Ni, raises the formation energy of AlCoCrFeNi HEA (see Fig. 3c, and the blue curve with squares in Fig. 3f). The effect of Fe is like that of Cr (see Fig. 3d, and the black curve with cross marks in Fig. 3f), but it is comparatively weaker. The effect of Co on the formation energy is shown in Fig. 3e. Contrary to other principal elements, the optimal value of the molar fraction of Co that leads to the lowest formation energy is located within a narrow range between 17 and 22% of Co (see the orange curve with diamonds in Fig. 3f). Similar results were obtained for the effect of molar fraction of the principal elements on the formation energy of the AlCoCrFeNi HEA with a BCC lattice (see Supplementary Fig. S2).

### Mass density

The density of the AlCoCrFeNi HEA with a BCC lattice is plotted vs the molar fraction of Al and Cr in a three-dimensional plot as shown in Fig. 2d (see also Supplementary Fig. S1 for FCC lattice), where the molar fraction of Ni is indicated by marker color, and the molar fraction of Co by marker size. The density manifold is represented by an inclined plane for both the FCC and BCC lattices.

**Figure 4:**
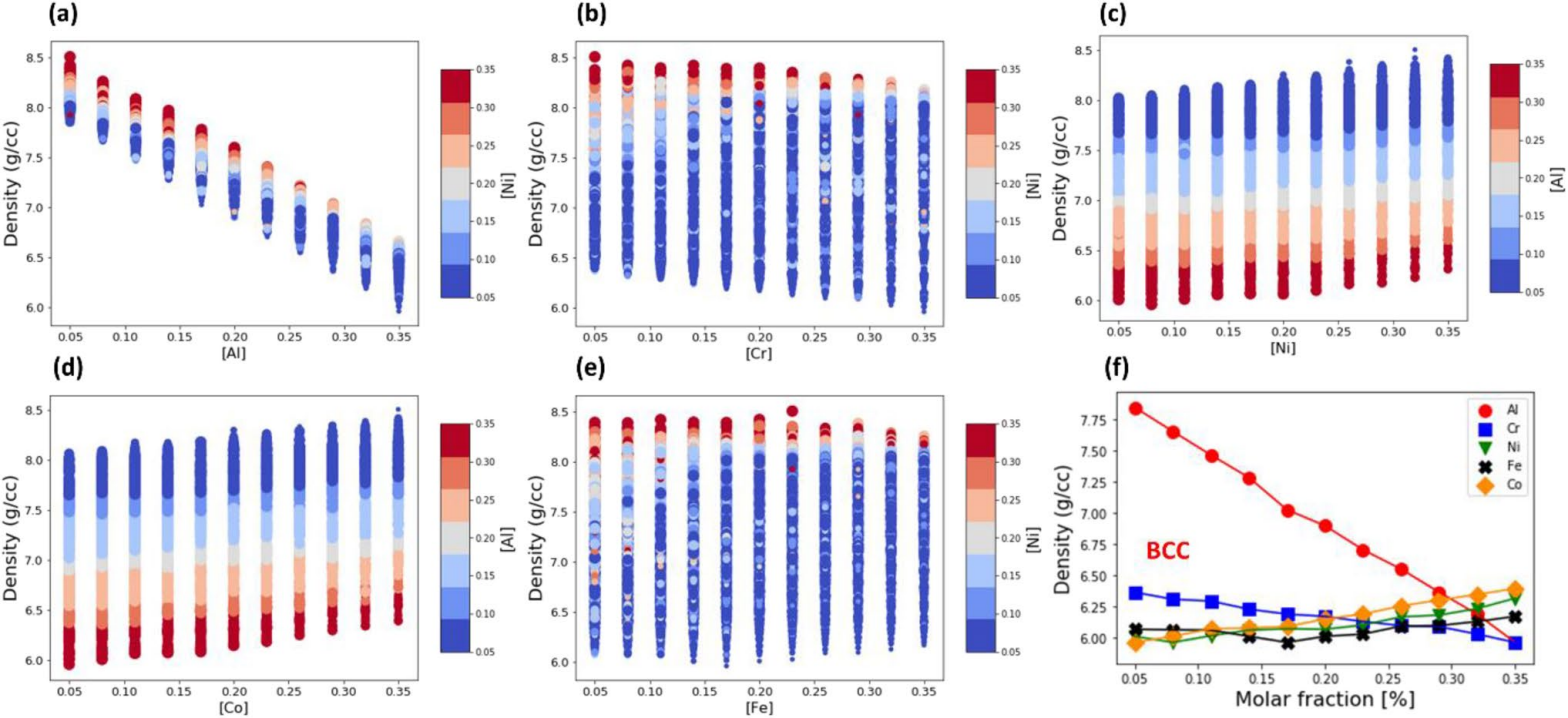
The effect of molar fraction of constituent elements on the density of BCC AlCoCrFeNi HEA. The density is plotted vs. the molar fraction of Al (**a**), Cr (**b**), Ni (**c**), Co (**d**) and Fe (**e**). For a given HEA composition, marker color indicates the molar fraction of Ni in (**a**), (**b**), (**e**) and Al in (**c**), (**d**). Marker size is proportional to the molar fraction of Co in (**a**), (**b**), (**e**) and Cr in (**c**), (**d**). (**f**) The density minimum of AlCoCrFeNi HEA with BCC lattice as a function of molar fraction of constituent elements: Al (red circles), Cr (blue squares), Ni (green triangles), Fe (black crosses) and Co (orange diamonds).

Figure 4 plots the density of AlCoCrFeNi HEA with BCC lattice against a molar fraction of the constituent elements (see also Supplementary Fig. S3 for FCC lattice). The density of the HEA compositions with the same molar fraction of a specific element is plotted as a stacked column. Marker color and size indicate the molar fraction of two other principal elements. The density minimum (bottom of each stacked column) as a function of the molar fraction of the principal element is plotted in Fig. 4f.

It can be seen in Fig. 4a that the lightest element, Al, has the strongest effect on the density, which decreases linearly with an increase in the molar fraction of Al (see the red line with circles in Fig. 4f). The effect of Cr is like that of Al, as shown in Fig. 4b, the density decreases with an increase in the molar fraction of Cr. Yet, in comparison with Al, the effect of Cr is substantially weaker (see the blue line with squares in Fig. 4f). In contrast to Al and Cr, the density of the AlCoCrFeNi HEA rises with an increase in the molar fraction of Ni (see Fig. 4c, and the green line with triangles in Fig. 4f) and Co (see Fig. 4d, and the yellow line with diamonds in Fig. 4f). However, the effect of Co is stronger than that of Ni. The effect of Fe is more intricate in comparison with other constituent elements as shown in Fig. 4e: The density increases at the low and high molar fractions of Fe, while its minimum is in the narrow range between 15 and 20% of Fe (see the black line with crosses in Fig. 3f). Similar results were found on the effect of constituent elements on the density of the AlCoCrFeNi HEA with an FCC lattice (see Supplementary Fig. S3).

### Formation energy vs mass density

**Figure 5:**
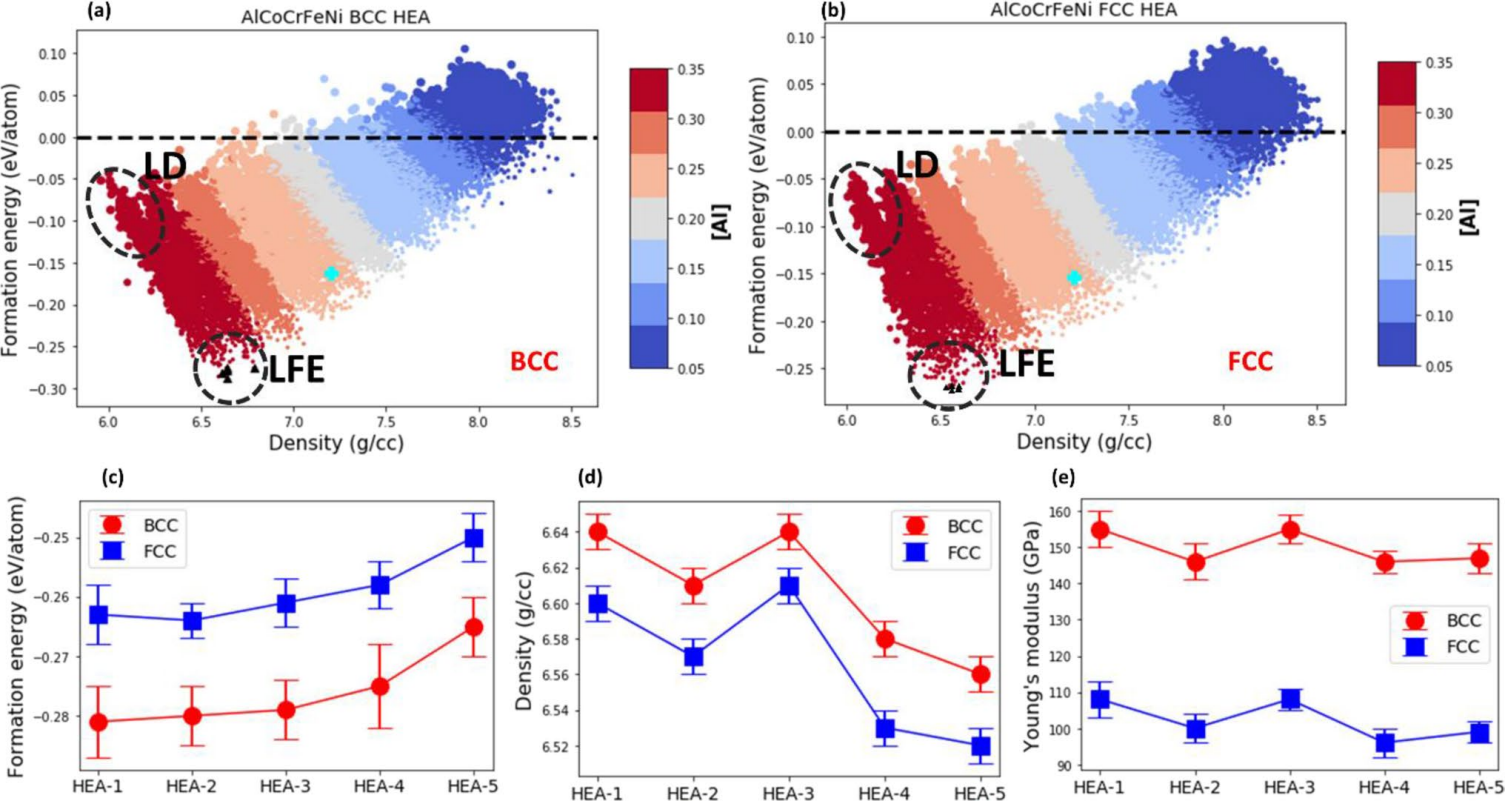
(**a**), (**b**) The calculated formation energy per atom vs. the mass density for AlCoCrFeNi HEA with BCC (**a**) and FCC (**b**) lattice structure. Marker color indicates the molar fraction of Al and marker size the molar fraction of Cr (**b**). Closed dashed lines outline the region of compositions with low densities (LD), and the region of compositions with low formation energies (LFE). The top five most energetically stable compositions are indicated by black triangles. The equimolar composition is indicated by cyan cross symbol (**c**)-(**e**) The formation energy per atom (**c**), mass density (**d**), and Young’s modulus (**e**) for the top five most energetically stable compositions of AlCoCrFeNi HEA with BCC lattice (red circles). The selected [AlCoCrFeNi] compositions are HEA-1: [0.35,0.22,0.05,0.05,0.33], HEA-2: [0.35,0.17,0.05,0.08,0.35], HEA-3: [0.35,0.23,0.05,0.05,0.32], HEA-4: [0.35,0.15,0.05,0.11,0.34] and HEA-5: [0.35,0.16,0.06,0.11,0.32]. The formation energy, density, and elastic modulus for the same compositions of AlCoCrFeNi HEA with FCC lattice structure (blue squares) are plotted for comparison.

The formation energies per atom vs the density for the AlCoCrFeNi HEA with BCC and FCC lattice are plotted in Fig. 5a and b, respectively. Marker color indicates the molar fraction of Al, and marker size the molar fraction of Cr in Fig. 5a, b. The formation energies per atom vs mass density with marker color indicating the molar fraction of Co, Cr, Fe and Ni for the AlCoCrFeNi HEA with BCC and FCC lattice structure are shown in Supplementary Figs. S5 and S6.

As can be seen in Fig. 5a, b, the higher the molar fraction of Al, the lower the formation energy and density (see the low density (LD) and the low formation energy (LFE) regions in Fig. 5a, b).

Similarly, the higher the molar fraction of Cr, the lower the density of the AlCoCrFeNi HEA (see the LD region in Fig. 5b). However, in contrast to Al, the lower the molar fraction of Cr, the lower the formation energy (see the LFE region in Fig. 5b).

There is a positive correlation between the formation energy and density as illustrated in Fig. 5a, b. Nevertheless, the set of compositions with low formation energies (see the LFE region in Fig. 5a, b) and the set of low-density compositions (see the LD region in Fig. 5a, b) do not intersect. Therefore, an adequate compromise between the low formation energy and the low density should be reached in the design of AlCoCrFeNi HEAs. Although for some constituent elements, such as Al, both the formation energy and the density of AlCoCrFeNi HEA can be lowered by increasing its molar fraction, for other constituent elements, such as Cr and Ni, one can either lower the density or formation energy by maximizing (or minimizing) molar fraction of the selected element. Therefore, the ultimate design of a AlCoCrFeNi HEA for specific applications requires finding a suitable compositional combination.

### The top five most energetically stable compositions of AlCoCrFeNi HEA

Finally, the top five most energetically stable compositions of the AlCoCrFeNi HEA with BCC and FCC lattice are selected for further analysis. The selected five compositions for the BCC lattice are reported in Table 1, and their formation energy, mass density, and Young’s modulus are shown in Fig. 5 (red circles), where, for comparison, these properties for the same compositions of AlCoCrFeNi with an FCC lattice are provided (blue squares). It is seen from Table 1 that each composition contains the relatively high molar fraction of Al and Ni (~ 35%), a moderate fraction of Co (~ 15–23%), and comparatively low fraction of Cr and Fe (~ 5%). It is found that the formation energies of the top five compositions of AlCoCrFeNi with a BCC lattice are lower than those with the same compositions with an FCC lattice, even though the difference is comparatively small (see Fig. 5c). The mass density of the top-five compositions of the AlCoCrFeNi HEA with a BCC lattice is marginally higher than those with an FCC lattice (see Fig. 5d). The calculated elastic moduli (see Fig. 5e for Young’s modulus and Supplementary Table S2 for bulk, and shear moduli) are noticeably larger than these of the same compositions but in FCC lattice.

The top five most energetically stable compositions with an FCC lattice are listed in Supplementary Table S2. It is found that the formation energies for the same compositions of the AlCoCrFeNi HEA with a BCC lattice are lower (see Supplementary Table S2), and the values of elastic moduli are higher (see Supplementary Table S3). Therefore, the top-five most energetically stable compositions of the AlCoCrFeNi HEA with a BCC lattice are best suited for applications compared to those in FCC lattice.

## Discussion

### Validation of the PSSOS method

**Figure 6:**
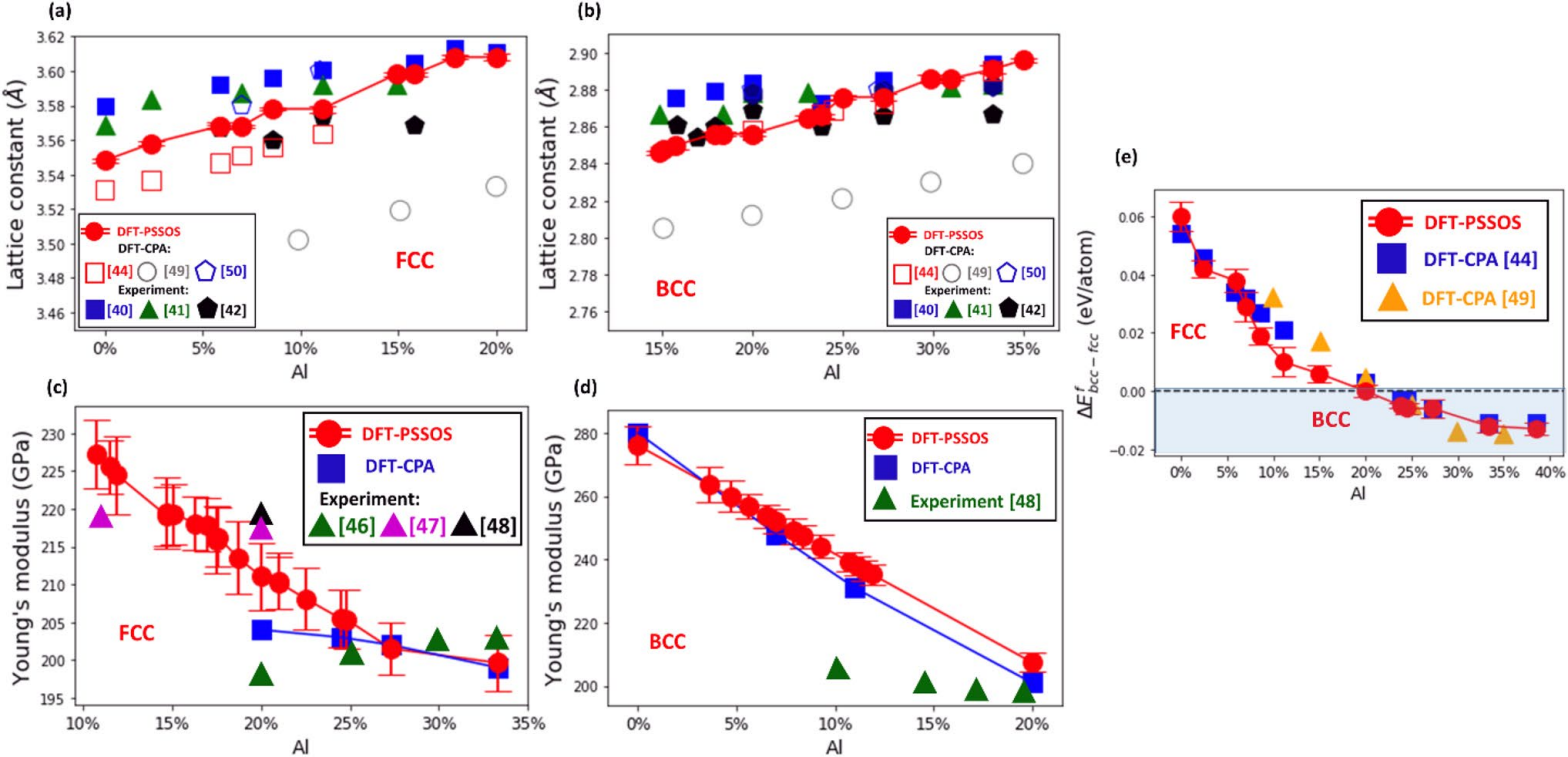
(**a**), (**b**) Lattice constant of Al_x_(CoCrFeNi)_1-x_ in FCC (**a**) and BCC (**b**) phase vs. molar fraction of Al. The values of lattice constant obtained by the PSSOS method are represented by red circles; the error-bars represent standard deviations, and the red line is a guide to the eye. The experimental measurements are taken from Chou et al.^40^ (blue squares), Wang et al.^41^ (green triangles), and Zhu et al.^42^ (black pentagons), while the values obtained by CPA-based DFT calculations are taken from Jasiewicz et al.^44^ (open circles), Tian et al.^49^ (open squares), and Leong et al.^50^ (open pentagons). (**c**), (**d**) The Young’s modulus of Al_x_(CoCrFeNi)_1-x_ in FCC (**c**) and BCC (**d**) phase is plotted against molar fraction of Al. The values of elastic modulus calculated by the PSSOS method (red circles) are compared with those calculated by the CPA-based DFT method^49^ (blue squares). The experimental measurements are taken from Li et al.^46^ (green triangles), Jiao et al.^47^ (orange triangles) and Mohanty et al.^48^ (magenta triangles) (**e**) Difference between the formation energy per atom of Al_x_(CoCrFeNi)_1-x_ in BCC and FCC phase as a function of molar fraction of Al. The energy differences, $\Delta E_{bcc - fcc}^{f}$, obtained by the PSSOS method are represented by red circles, the $\Delta E_{bcc - fcc}^{f}$ obtained by the CPA-based DFT calculations are taken from Jasiewicz et al.^44^ (orange triangles) and Tian et al.^49^ (blue squares). The dashed line separates the region with $\Delta E_{bcc - fcc}^{f} \ge 0$, where the FCC phase is more stable than BCC, and $\Delta E_{bcc - fcc}^{f} \le 0$ where the BCC phase is more stable than FCC.

To validate the PSSOS method, we compared the type of stable phases obtained by the PSSOS method for a given AlCoCrFeNi composition with the experimentally measured ones^40–43^ and the predicted ones by DFT based on the CPA approximation^44,45^. In addition, the corresponding lattice constants and Young’s modulus were calculated and compared (see Fig. 6).

In experiments, the molar fraction of a single constituent element, typically, Al is usually varied over a specific range as in a set of Al_x_(CoCrFeNi)_1-x_ compositions. For each molar fraction, *x*, of Al, one determines the most stable phase (FCC, BCC, or both) and measures the resultant lattice constant. First, we compared the phases predicted by the PSSOS method with those observed experimentally^40–43^ for Al_x_(CoCrFeNi)_1-x_ compositions. It was found that they are in good agreement for the single phase AlCoCrFeNi HEAs. At the low molar fraction of Al ($\le$ 20%), the FCC phase is stable, while at the high molar fraction of Al ($\ge$ 20%), the BCC phase is stable. Next, we compared the values of lattice constant for Al_x_(CoCrFeNi)_1-x_ compositions calculated by the PSSOS method with the experimentally measured values and values calculated by the CPA-based DFT method for both FCC and BCC phases (see Fig. 6a, b). As can be seen in Fig. 6a, b, the lattice constants obtained by the PSSOS method correspond well with experimental measurements and calculated results from CPA. Similarly, we observe that the lattice constants for Ni_x_(AlCoCrFe)_1-x_ (where the molar fraction of Ni is varied) are in a close agreement with the experiment and CPA results too (see Supplementary Fig. S7).

For more precise validation of the PSSOS method, we obtained the difference between the formation energy of AlCoCrFeNi with BCC and FCC lattice for the set Al_x_(CoCrFeNi)_1-x_ compositions, and compared the results with those from CPA-based DFT calculations^44,45^ (see Fig. 6e). It should be noted that when the energy difference is positive, the FCC phase is more stable than BCC (and vice versa for the negative energy difference). As can be seen in Fig. 6c, both the predicted energetically stable phases and energy differences between BCC and FCC phases calculated by the PSSOS method and the CPA-based DFT method agree well each other, thus providing additional support for the validity and accuracy of the PSSOS method.

Finally, we compared the values of Young’s modulus calculated by the PSSOS method and the CPA-based DFT method^45^ for the set Al_x_(CoCrFeNi)_1-x_ compositions (see Fig. 6c, d). As can be seen in Fig. 6c, d, both the PSSOS and CPA methods predict the value of elastic module for AlCoCrFeNi HEA in solid solution state with a comparable accuracy, as compared with the experimental measurements taken from Li et al.^46^, Jiao et al.^47^ and Mohanty et al.^48^ The results obtained from the proposed method are in line with the CPA-based DFT methods. The overall agreement in values for lattice constant, formation energy and elastic modulus calculated by the PSSOS method and those obtained experimentally or by the CPA-based DFT add considerable support for the validity of PSSOS. According to Fig. 6c, d, the results obtained by the PSSOS method are close to those obtained by the CPA. This is because the HEA contains atoms with similar atomic radius and electronic configuration, such as Fe, Co, Ni, and Cr combined with Al9. In this case, the CPA or VCA method can be used to reduce computational cost. But in general, if an HEA contains atoms with quite different atomic radius and electronic configuration, such as Ti, Nb or Be, there is the apparent lattice distortion in the SOS structures, and the results obtained by the PSSOS method are more accurate than those obtained by the mean-field based CPA or VCA^26,33^.

### Computational efficiency of the present method

It is well-known that the computational cost (*CC*) of DFT calculations scales with the $\sim$ 3rd power of the number of atoms, *N*, that is,$CC\sim O(N^{3} )$. For an equimolar quinary HEA, an SQS sample typically contains *N* $=$ 125 atoms, the computational cost of the SQS or CPA-based DFT calculations is $CC\sim O(125^{3} ) = O(10^{6} )$. In contrast, the computational cost of the PSSOS method scales as $CC\sim O(s \cdot n \cdot N^{3} )$, where *s* is the number of SSOS solutions, *n* is the number of SOS per set, and *N* is the number of atoms per SOS. Consequently, for SSOS with *s* = 5, *n* $\sim$ 30 and N $\sim$ 7, the $CC\sim O(5 \cdot 30 \cdot 7^{3} ) = O(10^{5} ).$ Hence, for the equimolar case, the PSSOS method is an order of magnitude more computationally efficient than the SQS method. However, for non-equimolar HEAs with large composition disparities, at least a thousand atoms are required for the SQS method. For such cases, the PSSOS method is more computationally efficient by several orders of magnitude than the SQS method. Hence, compared to the SQS method, the PSSOS method is comparable in accuracy, but considerably more computationally efficient, and thus suitable for HF, FT computations for HEAs.

## Method

### The SSOS method

The key idea of the SSOS method is to use a set of special ordered structures to model HEAs. In this method, the complete set of SOS with cubic lattice structure are first constructed. A small set of SOS is selected to match exactly the target atomic pair-correlation functions of a given HEA composition and lattice structure. Then, the geometry of the selected SOS is optimized by DFT, and the corresponding properties, like formation energy, lattice constant, density, and elastic moduli, are calculated as the weighted average over the properties of the selected SOS. Hence, the main task is to identify the set of SOS and the corresponding weights^26,33^. The chief advantage of the SSOS method is the substantial reduction in the number of atoms required to model an HEA in the ideal solid solution phase, since the selected SOS samples in principle contain only a few atoms. As a result, its computational cost is considerably smaller than that of SQS, making it preferable for HT DFT calculations. Moreover, any non-equimolar composition can be accurately modelled with the SSOS method using small structures, whereas SQS models for most non-equimolar compositions require many atoms to satisfy the periodic boundary conditions, thus making HT DFT-based SQS calculations impractical. For example, to model a non-equimolar composition of AlCoCrFeNi HEA in BCC lattice $\left[ {\frac{7}{20},\frac{11}{{50}},\frac{1}{20},\frac{1}{20},\frac{33}{{100}}} \right]$ by the SQS method with the smallest possible non-orthogonal equal-side sample, one needs to use 10^3^ = 1000 atoms. To accurately construct such an SQS sample, one typically needs to find the least common denominator (for the molar fractions), the cube root of which is an integer (with the smallest value). If one multiplies the three different denominators (20, 50 and 100) by a set of unknown positive integers $N_{1} , N_{2}$ and $N_{3}$, then three equations for the common denominator are obtained: *20* $N_{1}$ = $2^{2} 5^{1} N_{1} = M^{3}$, *50* $N_{2}$ = $2^{1} 5^{2} N_{2} = M^{3} ,{ }$ and *100* $N_{3}$ = $2^{2} 5^{2} N_{3} = M^{3}$. The solution of these equations requires the use of the unknowns which complement the products to the 3^rd^ power. Thus, $N_{1} = 2^{1} 5^{2} = 50, N_{2} = 2^{2} 5^{1} = 20$ and $N_{3} = 2^{1} 5^{1} = 10.$ The obtained solution leads to the smallest possible SQS sample with $M^{3} = 10^{3}$ = 1000 atoms.

### Calculation of pair correlation functions in solid solution state

The atomic pair correlation functions quantitatively describe the neighborhood of the atom of interest. They indicate the number and type of neighboring atoms located around the atom up to a specific range. In the ideal solid solution phase, these pair correlation functions can be obtained analytically. For a given composition of AlCoCrFeNi HEA, we count the fraction of all possible pairs Al–Al, Al–Co, Co–Co, Co–Cr, Co–Fe, …, Ni–Ni formed by atoms separated up to 1st, 2nd, and 3rd nearest neighbor (NN) distance. For the ideal solid solution phase of an equimolar HEA, the fraction of pairs formed by the same elements is constant (which does not depend on NN distance) and is equal to half of that formed by two different elements ^35^. The fraction of specific pairs, for example, Al-Co pairs in the ideal solid solution phase, is obtained by counting the number of pairs formed by selected atoms. One can make two pairs (Al-Co and Co-Al) by using Al and Co atoms, but only one pair out of Al atoms (Al-Al) or Co atoms (Co–Co). For an equimolar composition of quinary HEA, the probability of selecting an Al atom (or any constituent element) is the same, that is, *p* = $\frac{1}{5}$, as the molar fraction of Al, *c*(Al). Thus, the fraction of Al-Co pairs is P = $2 \cdot c\left( {Al} \right) \cdot c\left( {Co} \right)$ = $2 \cdot \frac{1}{5} \cdot \frac{1}{5}$ = 0.08. But if the same elements are used, the fraction of Al-Al (or Co–Co) is only P = $c\left( {Al} \right) \cdot c\left( {Al} \right)$ = $\frac{1}{5} \cdot \frac{1}{5}$ = 0.04 (see pair correlation functions of equimolar HEA in Supplementary Fig. S8a of Supplementary Materials).

For a given HEA with non-equimolar composition, the corresponding pair correlation functions are calculated in the same way. For example, the fraction of Al-Al pairs is given by P = $c\left( {Al} \right) \cdot c\left( {Al} \right)$ and Al-Co pairs is P = $2 \cdot c\left( {Al} \right) \cdot c\left( {Co} \right)$, that is, only the probability to select a specific element is equal to its molar fraction, which is specified by its non-equimolar composition^35^ (see an example of pair correlation functions for a non-equimolar HEA in Supplementary Fig. S8b of Supplementary Materials).

### Construction of SOS samples

Next, we construct the SOS samples with FCC and BCC lattices. In the SSOS method, symmetry-unique SOS are constructed by selecting three independent lattice vectors describing a non-orthogonal and non-primitive (non-conventional) unit cell of cubic lattice (see Fig. 1b). The positions of basis atoms $R_{\alpha }^{i}$ located within the selected unit cell are given by: $R_{\alpha }^{i} = \sum\nolimits_{K}^{3} {\zeta_{j}^{i} a_{\alpha }^{K} }$. They are expressed relative to the selected non-primitive lattice vectors $a_{\alpha }^{j}$, by using fractional coordinates $\zeta_{j}^{i}$ satisfying the conditions: ($0 \le \zeta_{j}^{i} < 1)$, where $K = 1, 2, 3$ is the index of the lattice vectors $, \alpha = x, y, z$ are projections on Cartesian axes, and $i = 1, 2, 3, \ldots , N_{at}$ indicates the atoms within the SOS. The complete set of $N_{at} =$ 5-atom SOS and a partial set of $N_{at} =$ 6-atom SOS for FCC lattice are given in citations^26,33^. In order to construct a set of all possible SOS, the TTK package^51^ was used. The five constituent elements (Al, Co, Cr, Fe and Ni) were distributed based on atom sites $R_{\alpha }^{i}$ of the constructed SOS. For every SOS, we calculated the corresponding atomic pair-correlation functions, {$\phi_{i} \}$ up to 3rd NN range. The obtained pair-correlation functions are the unique “fingerprints” identifying the specific SOS with FCC or BCC lattice structure. In total, we found a very large set of 5-, 6- and 7-atom SOS whose atomic pair-correlations can be used to model HEAs with non-equimolar compositions. For the SOS set with FCC lattice, the number of 5-atom SOS is $N_{5}$ = 1614, 6-atom SOS is $N_{6}$ = 13,685 and 7-atom SOS is $N_{7}$ = 29,775, giving in total $N_{fcc} =$ 45,074. Similarly, for BCC lattice, the number of SOS containing 5 atoms is $N_{5}$ = 1,614, 6-atom SOS is $N_{6}$ = 14,560 and 7-atom SOS is $N_{7}$ = 35,665, giving in total $N_{bcc} =$ 51,839.

### The preselected SOS subset

Obtaining an SSOS solution from the complete set of SOS (containing $\sim$ 50,000 samples) and then optimizing their geometries and calculating their characteristic properties by DFT is prohibitively expensive. To overcome this difficulty, we identify $\sim$ 1000 of the most frequently used SOS from the complete set and then look for the SSOS solutions by taking SOS only from this small DFT preselected SOS subset.

When the preselected SOS set is used, the number of SOS per SSOS solution can be larger compared to the case when the SOS cases are taken from the complete set, since only a fraction of all the SOS are used. Thus, the number and the type of all available pair correlation functions are limited. In practice, we start with a trial set containing up to *n* = 34 SOS (as in the case when the pair correlation functions match the target functions up to 3rd NN range^33^ and the complete set of SOS is used) and then continue to increase the number of SOS per set until an SSOS solution can be identified. We found that, on average, the number of SOS per SSOS solution is noticeably larger (around *n* $\sim$ 60–90). Yet, since all the SOS in the obtained SSOS solution are taken from the same preselected subset, the computational cost of the set expansion is negligible. We verified the new approach by comparing the identified SSOS solutions for a given HEA composition with those obtained by the established SSOS method and found that the results from the PSSOS approach are practically identical.

### Identification of the SSOS solutions by matching the pair correlation functions

Our subsequent task is to select a minimal number subset of SOS ($n <$ $N_{tot}$) from the preselected set of SOS, which constitutes an SSOS solution. To do so, we identify the corresponding weights for a linear combination of their pair correlation functions ${\Phi } = \sum\nolimits_{i}^{n} {w_{i} \phi_{i} }$, which precisely match the target pair correlation functions ${\Phi }$ up to the 3rd NN range. The selection of a specific SOS subset and the corresponding weights is a two-step optimization process: First, a trial subset with a selected number of SOS, $n,$ is chosen from the preselected SOS set, and then the weight coefficients are found by linear regression. The set of weights $\{ w_{i}$} that best reproduces the target pair correlation functions$,$ is obtained by minimizing the residual coefficient $\lambda = \left( {{\Phi } - \sum\nolimits_{i}^{n} {w_{i} \phi_{i} } } \right)^{2} .$ The linear regression is used to calculate both the weights $\{ w_{i}$} and $\lambda$. The accuracy of the linear regression is measured by λ, which is the key condition for accepting a selected trial subset of SOS as an SSOS solution. For an SSOS solution to be accepted, a minimal value of λ < 10^–12^ was used.

In view of the prohibitively large number of SSOS solutions, a direct exhaustive search for all the SSOS solutions is impractical. Instead, we used a stochastic search, namely the hill climbing method (HCM)^39^, to minimize $\lambda = \left( {{\Phi } - \sum\nolimits_{i}^{n} {w_{i} \phi_{i} } } \right)^{2} .$ Since the HCM is a local search algorithm, there is a possibility that due to the rugged solution landscape, {$\phi_{i} \}$, the search may end up in a local minimum of λ and miss out the global minimum $\left( {{\uplambda } = 0} \right)$. Thus, to explore the solution landscape thoroughly, the HCM routine was restarted multiple times at different locations of the SOS space to search thoroughly, albeit stochastically through it. However, since it is not possible to obtain all the SSOS solutions for a given HEA with non-trivial target pair correlation functions, we only search for a finite number of SSOS solutions. With the HCM method, we were able to identify a large number of SSOS solutions with the maximal number of *n* = 100 SOS and the minimal number of *n* = 34 SOS^26,33^ when the target pair-correlation functions of the HEA with SRO are matched up to the 3rd NN range. It is possible to go beyond the 3^rd^ NN range, but the gain in the accuracy of the calculated properties, which was estimated by going from the 2nd to the 3rd NN range, is less than < 1%^34^. Therefore, the minor improvement in the accuracy does not offset the substantial increase in the computational cost of constructing and optimizing additional SOS required to match the target functions up to the 4th (or higher) NN range.

Here, we only considered matching of the pair correlation function; thus, one might think that such matching may skew against the effect of triplet correlations and result in an unrepresentative structure for HEAs. However, we found that when the target pair correlation functions are matched with the highest accuracy, the discrepancy between the corresponding triplet correlation functions and above is negligibly small^34^.

### Calculation of the HEA properties

To deal with the non-uniqueness of the SSOS solutions, we calculated the properties of the HEA as a simple average over a subset of the obtained SSOS solutions^33^. Since all the acquired solutions match the target pair correlation functions with high precision, we used the triple correlation functions to select the subset of the best SSOS solutions for the averaging procedure. The triple correlation functions were calculated for each SOS in the same way as the pair correlation functions^35^. All the obtained SSOS solutions were sorted according to the accuracy with which they match the target triple correlation functions of the ideal solid solution phase. The first top five solutions were selected as a subset of the SSOS solutions, over which we averaged the calculated properties of a HEA sample with a given composition.

The properties of the AlCoCrFeNi HEA (energy, lattice constant, density, and elastic moduli) for different compositions were calculated in two steps: First, a weighted average over SOS samples constituting a given SSOS solution, $s,$ was calculated, and then a simple average over the selected top five SSOS solutions was obtained. For example, the ground state energy $, E_{s} ,$ is calculated according to:$E_{s} = \mathop \sum \nolimits_{i}^{n} w_{i}^{s} E_{i}^{sos}$, where $w_{i}^{s}$ is the weight coefficient of the $i{\text{th}}$ SOS obtained for $s$-SSOS solution, $E_{i}^{sos}$ is the energy of the $i{\text{th}}$ SOS, and *n* is the number of SOS. The ground state energy of a given HEA, $\left\langle E \right\rangle$, is then obtained as $\left\langle E \right\rangle = \frac{1}{m} \mathop \sum \nolimits_{s}^{m} E_{s}$, where *m* is the number of the subset of SSOS solutions used for the averaging procedure (*m* = 5 in our case). The other properties (lattice constant, mass density, elastic moduli, and Poisson’s ratio) were calculated in the same way.

The formation energy of AlCoCrFeNi HEA with a given composition [$c\left( {Al} \right),c\left( {Co} \right),c\left( {Cr} \right),c\left( {Fe} \right),c\left( {Ni} \right)$] was calculated in the following way: We first obtained ground state energy per atom, < *E* > */N*, of AlCoCrFeNi HEA by the SSOS method, and then subtracted from it the energy (per atom), $E_{ROM} ,$ calculated according to the rule of mixtures: $E_{ROM} = c\left( {Al} \right)E_{Al} + c\left( {Co} \right)E_{Co} + c\left( {Cr} \right)E_{Cr} + c\left( {Fe} \right)E_{Fe} + c\left( {Ni} \right)E_{Ni}$, where $E_{Al}$ and $E_{Ni}$ are the ground state energies per atom for pure Al and Ni with FCC lattice, while $E_{Cr} ,$ $E_{Fe}$ are the ground state energies per atom for pure Cr and Fe with BCC lattice, respectively, and $E_{Co}$ is the ground state energy per atom for pure *Co* with HCP lattice.

### Systematic examination of the composition grid

We systematically explored the composition space of AlCoCrFeNi HEA by the PSSOS method. We selected the lower (5%) and the upper (35%) limits for the molar fractions of every constituent element of AlCoCrFeNi HEA. The increment in the molar fraction (grid step) was set to $\Delta =$ 3%. Although a quinary HEA contains five constituent elements, only four out of five molar fractions of the constituent elements are linearly independent, since the sum of them is equal to one: $c\left( {Al} \right) + c\left( {Co} \right) + c\left( {Cr} \right) + c\left( {Fe} \right) + c\left( {Ni} \right) = 1.$ Therefore, in order to construct the composition grid of AlCoCrFeNi HEA, we systematically varied the molar fraction of Al, Co, Cr and Fe within the specified range, while the molar fraction of Ni was obtained as a dependent variable. With $\Delta =$ 3% increment in the molar fraction, we generated one equimolar and 8800 non-equimolar compositions (see Fig. 2). The formation energies and mass densities were calculated for all the generated compositions, and the top-five compositions with the lowest formation energy were selected for AlCoCrFeNi HEA with BCC and FCC lattices. With the top-five grid compositions being identified, we verified if the better solutions with lower formation energy can be found in the vicinity of these compositions. Therefore, we applied the HCM to explore locally the off-grid composition space around the top-five grid compositions. Three new compositions with the lower formation energy were found for both the FCC and BCC lattices (see Table 1 and Fig. 5, as well as Supplementary Table S1 and Supplementary Fig. S4).

### Details of the DFT calculations

All our DFT calculations were carried out with the generalized Perdew-Burke-Ernzerhof^52^ and the projector-augmented wave (PAW) pseudopotential plane-wave method^53^, as implemented in the VASP code^54^. For the PAW pseudopotentials, we included 2s^2^3p^1^, 3p^6^d^7^4s^2^, 3d^5^4s^1^, 3p^6^d^8^4s^2^, and 3p^6^d^6^4s^2^, as valence electrons for Al, Co, Cr, Ni, and Fe, respectively. We also calculated the elastic constants by deforming the SQS and SOS samples and deriving their elastic constants from the strain–stress relation. The corresponding isotropic elastic moduli (Young’s, bulk and shear modulus) and Poisson’s ratio were derived from these elastic constants by using the Hill’s approximation scheme^55^.

For the SOS DFT calculations, we used 12 × 12 × 12 Monkhorst − Pack^56^ k-point grid for unit cell geometry optimizations and energy calculations, and a plane-wave basis set with an energy cut-off of 520 eV was adopted. Good convergence was obtained with these parameters, and the total energy was converged to 10^−7^ eV per atom. Spin polarization was considered in this study. Spin polarization was considered in this study. In our calculations we started with relatively large initial local magnetic moments, because in some cases, the default values might not be sufficiently big to properly describe the effect of interaction of the spin magnetic moments. For our spin polarized collinear DFT calculations we specified the following initial magnetic moment: M = 0.6μ_B_ for each Al atom, and M = 5μ_B_ for each Co, Cr, Fe, and Ni atom. The energy minimization was performed using a conjugate-gradient algorithm to relax the ions into their instantaneous ground state without constraining lattice constants.

## Supplementary Information

Supplementary Information.

## Data Availability

The datasets used and/or analyzed during the current study is available from the corresponding author on request.
