# Supplementary material for: A first-principles-based high fidelity, high throughput approach for the design of high entropy alloys
Source: Sci Rep. 2022 Jul 13;12:11894. doi: 10.1038/s41598-022-16082-w (PMC9279411; doi:10.1038/s41598-022-16082-w)
Supplement: Supplementary file 1 — Supplementary Information. [file 41598_2022_16082_MOESM1_ESM.docx]

# **Supplementary Materials**

# **A First-principles-based high fidelity, high throughput approach for the design of high entropy alloys**

**V. Sorkin^*^, Z.G. Yu, S. Chen, Teck L. Tan, Z.H. Aitken, and Y.W. Zhang^*^**

Institute of High Performance Computing, A*STAR, Singapore 138632, corresponding author e-mails: sorkinv@ihpc.a-star.edu.sg and zhangyw@ihpc.a-star.edu.sg

# **Formation energy** **of the AlCoCrFeNi HEA with BCC and FCC lattice structure**

The calculated formation energy per atom and mass density of the AlCoCrFeNi HEA with an FCC lattice structure are plotted vs. molar fraction of Al and Cr in a three-dimensional plot as shown in  Figure S1. Since the composition space of the AlCoCrFeNi HEA is described by four independent molar fractions, we indicate the molar fraction of Ni by marker color and molar fraction of Co by marker size. The formation energy manifold (see Figure 1 (a)) is represented by an inclined plane, with the most energetically stable compositions with the lowest formation energy located at the left corner.


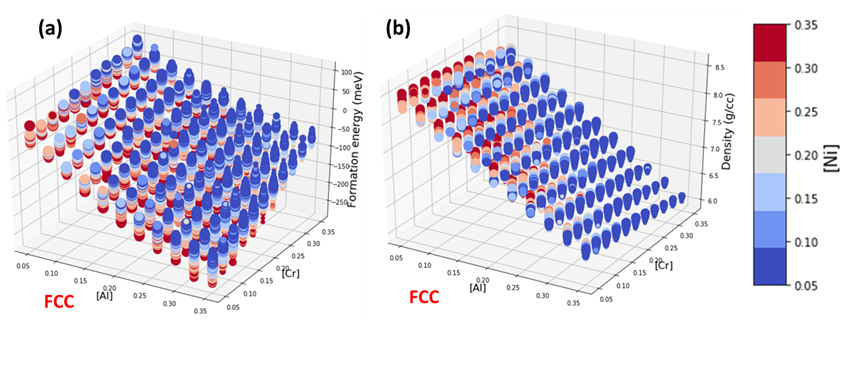


**Supplementary Figure S1:** Formation energy per atom (a) and mass density (b) of the AlCoCrFeNi HEA with an FCC lattice structure vs. molar fraction of Al and Cr. Marker color indicated molar fraction of Ni, while marker size corresponds to molar fraction of Co for a given HEA composition

In  Figure S2, we plot the formation energy per atom vs. molar fraction of constituent elements of the AlCoCrFeNi HEA with a BCC lattice. The formation energies of HEA compositions with the equal same molar fraction of one of their constituent elements are represented by a stacked vertical column. The size and color of markers indicate the molar fractions of two others principal elements (indicated in the color bar and caption). The bottom of each vertical column corresponds to the minimum of formation energy at a given molar fraction (see Figure S2 (f)).

As shown in Figure S2, the formation energy of the AlCoCrFeNi with a BCC lattice is apparently strongly affected by the molar fraction of specific constituent elements. For example, an increase in the molar fraction of Al results in practically linear reduction of the formation energy as shown in  Figure S2 (a) (see also the red curve with circles in Figure S2 (f)). Similarly, an increase in the molar fraction of Ni (see the green line with triangles in Figure S2 (f) and Figure S2 (d)) reduces the formation energy. Yet, the effect of Ni is significantly weaker than that of Al: the mean slope for Ni in  Figure S2 (f) is considerably smaller than that of Al. In contrast to Al and Ni, an increase in the molar fraction of Cr raises the formation energy of the AlCoCrFeNi HEA (see the blue curved line with squares in Figure S2 (f) and Figure S2 (b)). Likewise, an increase in molar fraction of Fe raises the formation energy of AlCoCrFeNi (see the black curve with cross marks Figure S2 (f) and Figure S2 (c)). However, in comparison with Cr, the effect of Fe is weaker. The effect of Co on the formation energy is shown in Figure S2 (e) (see also the yellow curved line with diamonds in Figure S2 (f)). Contrary to other constituent elements, the value of formation energy increases at low and high molar fraction of Co, while the minimal values lay in the intermediate range (from 15% to 25%) of the molar fraction of Co.


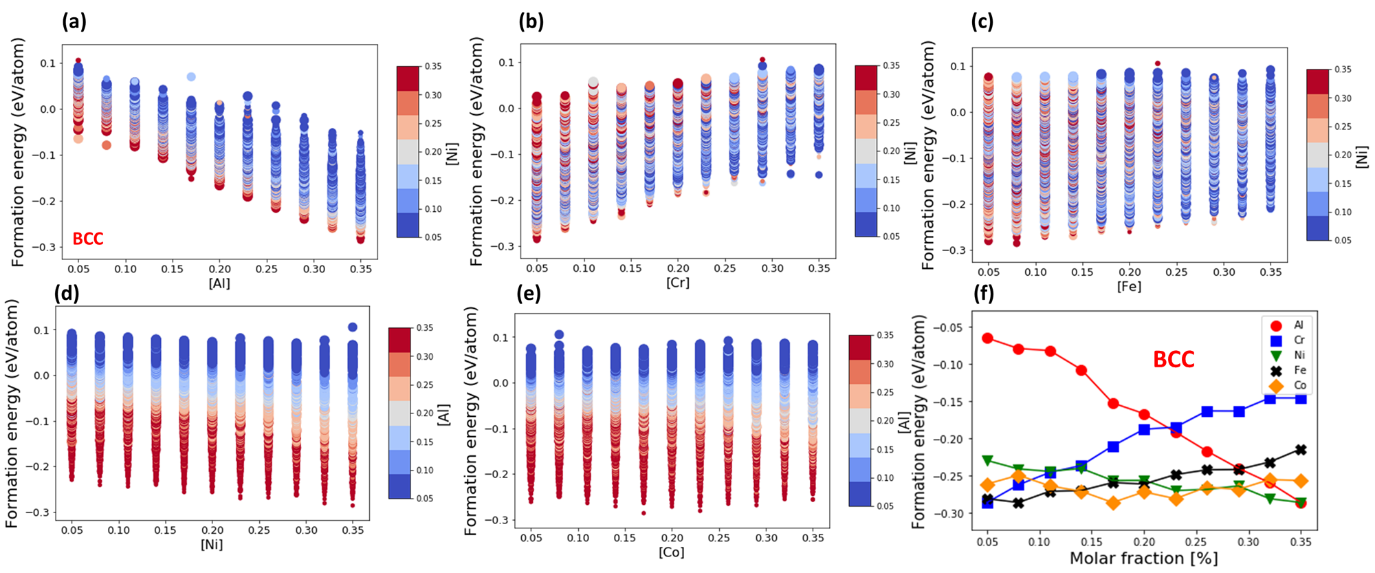


**Supplementary Figure S2:** The effect of molar fraction of constituent elements on the formation energy of the AlCoCrFeNi HEA with a BCC lattice. The formation energy (per atom) of AlCoCrFeNi HEA is plotted against the molar fraction of Al (a), Ni (b), Cr (c), Fe (d) and Co (e). For a given HEA composition marker color indicated the molar fraction of Ni in (a-c) and Al in (d, e). Marker size corresponds to the molar fraction of Co in (a, b, d) and Cr in (c, e). (f) The minimum of formation energy of the AlCoCrFeNi HEA with a BCC lattice as a function of molar fraction of the constituent elements: Al (red circles), Cr (blue squares), Ni (green triangles), Fe (black crosses) and Co (orange diamonds).

# **Mass density of the AlCoCrFeNi HEA with BCC and FCC lattice structure**

The mass density of the AlCoCrFeNi HEA in an FCC lattice is plotted against the molar fraction of Al as a three-dimensional plot in Figure S1 (b), where we specify the molar fraction of Ni by marker color, and the molar fraction of Co by marker size. As can be seen in Figure S1, the density manifold is represented by an inclined plane.

In Figure S3, we plot the mass density of the AlCoCrFeNi HEA in a BCC lattice vs. molar fraction of its constituent elements. The calculated densities of HEA compositions with equal molar fraction of a specific constituent element are plotted as a stacked vertical column. The color and size of markers indicate the molar fractions of two other principal elements. In Figure S3 (f), we plot the minimum of mass density (the bottom of each stacked column) as a function of molar fraction.

As can be seen in Figure S3 (a), the lightest element, Al, has the strongest effect: the density decreases nearly linearly with an increase in the molar fraction of Al (see the red line with circles in Figure S3 (f)). Similarly, the effect of Cr is like that of Al, as shown in Figure S3 (b), the density decreases with an increase in the molar fraction of Cr. However, in comparison to Al, the effect of Cr is markedly weaker (see the blue line with squares in Figure S3 (f)). In contrast to Al, the density increases with an increase in molar fraction of Ni (see Figure S3 (d), and the green line with triangles in Figure S3 (f)) and molar fraction of Co (see the yellow line with diamonds in Figure S3 (f), and Figure S3 (e)). The effect of Co on the density of AlCoCrFeNi is slightly stronger than that of Ni. The effect of Fe on the density of AlCoCrFeNi is more intricate in comparison with other constituent elements of AlCoCrFeNi as shown in Figure S3(c): the mass density increases when the molar fraction of Fe is too low or too high, while the minimum density is located between 15% and 20% (see the black curved line with crosses in Figure S3 (f)).


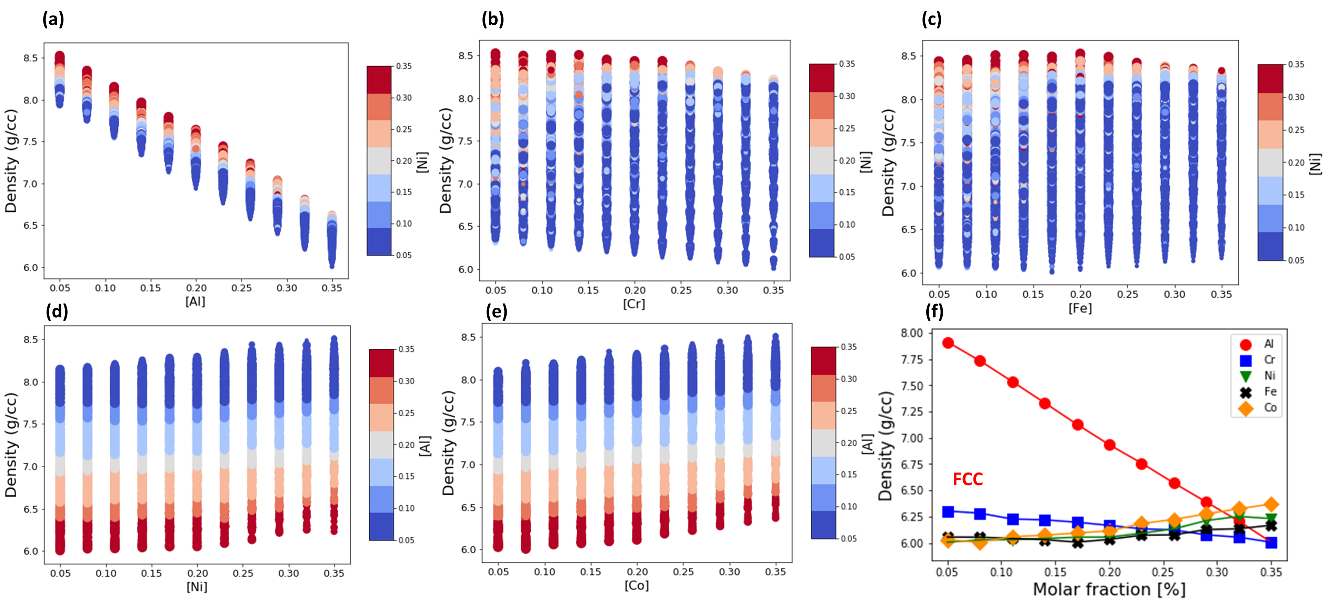


**Supplementary Figure S3:** The effect of molar fraction of constituent elements on the mass density of the AlCoCrFeNi HEA with an FCC lattice. The mass density of the AlCoCrFeNi HEA is plotted vs. molar fraction of Al (a), Ni (b), Co (c), Cr (d) and Fe (e). For a given HEA composition marker color indicated the molar fraction of Ni in (a-c) and Al in (d, e). Marker size corresponds to the molar fraction of Co in (a, b, d) and Cr in (c, e). (f) The minimum of mass density of the AlCoCrFeNi HEA with an FCC lattice as a function of molar fraction of the constituent elements: Al (red circles), Cr (blue squares), Ni (green triangles), Fe (black crosses) and Co (orange diamonds).

# **Top five BCC and FCC compositions of the AlCoCrFeNi HEA**

In this section, we report the values for the formation energy, mass density, lattice constant and elastic moduli calculated by the PSSOS method for top five most energetically stable compositions with the lowest formation energy (per atom) for the AlCoCrFeNi HEA with BCC and FCC lattice structures (see Supplementary Tables S1-3).

Each of the top-five compositions of the AlCoCrFeNi HEA with a BCC lattice contains the highest molar fraction of Al and Ni (35%), the lowest molar fraction of Cr and Fe (5%), and the intermediate molar fraction of Co (15%-23%). For comparison, for each calculated property of a given composition with a BCC lattice, we present the corresponding property for the same composition but with an FCC lattice. We found that the formation energies of all the top five compositions with a BCC lattice are lower than those with an FCC lattice, although the difference is comparatively small. The mass density of the top-five compositions of the AlCoCrFeNi HEA with a BCC lattice is marginally higher than those of the corresponding compositions with an FCC lattice. The calculated elastic moduli (Young’s, bulk, and shear moduli) for the AlCoCrFeNi HEA with a BCC lattice is markedly larger than those with the same composition but with an FCC lattice structure (see Table S1).

**Supplementary Table S1:** The lattice constants and elastic moduli of the top-five most stable compositions of the AlCoCrFeNi HEA with a BCC lattice as compared with those of the AlCoCrFeNi HEA with an FCC lattice with the same composition. The Poisson coefficient for top five compositions of the AlCoCrFeNi HEA with a BCC lattice is ν=0.22. Error bars indicate the standard deviation of averaging over a set of SSOS solutions.

| **AlCoCrFeNi composition** | **Lattice constant (Å)** | | **Bulk modulus (GPa)** | | **Shear modulus (GPa)** | |
| --- | --- | --- | --- | --- | --- | --- |
|  | **BCC** | **FCC** | **BCC** | **FCC** | **BCC** | **FCC** |
| [0.35,0.22,0.05,0.05,0.33] | 2.875±0.006 | 3.627 ± 0.006 | 133±2 | 110±1 | 92±2 | 74±2 |
| [0.35,0.17,0.05,0.08,0.35] | 2.871±0.005 | 3.624 ± 0.005 | 128±1 | 119±1 | 88±2 | 70±2 |
| [0.35,0.23,0.05,0.05,0.32] | 2.877 ± 0.003 | 3.629 ± 0.004 | 133±1 | 112±1 | 93±2 | 73±1 |
| [0.35,0.15,0.05,0.11,0.34] | 2.872±0.005 | 3.623 ± 0.004 | 129±1 | 117±2 | 89±2 | 67±2 |
| [0.35,0.16,0.06,0.11,0.32] | 2.877±0.005 | 3.632 ± 0.004 | 126±1 | 118±1 | 89±2 | 69±1 |

Likewise, each of the top five compositions of the AlCoCrFeNi with an FCC lattice structure contains the highest molar fraction of Al and Ni (35%), the lowest molar fraction of Cr and Fe (5%), and the intermediate molar fraction of Co (15%-23%). In Figure S4, we compare the values of the formation energy, mass density and Young’s modulus of the top five of AlCoCrFeNi with an FCC lattice with the values for the same AlCoCrFeNi compositions with a BCC lattice structure (see also Table S2 and Table S3). It can be seen from Figure S4 that for each of the top five compositions of AlCoCrFeNi with an FCC the corresponding composition with a BCC lattice structure has lower values of the formation energy, and higher values of the density and the elastic moduli.


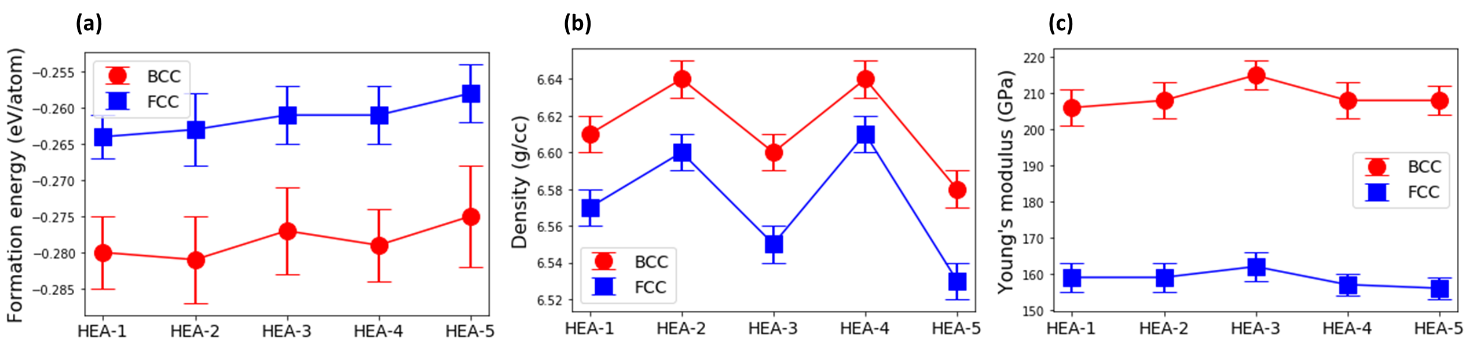


**Supplementary Figure S4:** The formation energy per atom (a), mass density (b) and Young’s modulus (c) for top-five most stable AlCoCrFeNi HEA compositions with an FCC lattice as compared with the same compositions of the AlCoCrFeNi HEA with a BCC lattice. The selected [AlCoCrFeNi] compositions are HEA-1: [0.35,0.17,0.05,0.08,0.35], HEA-2: [0.35,0.22,0.05,0.05,0.33], HEA-3: [0.35,0.17,0.05,0.09,0.34], HEA-4: [0.35,0.23,0.05,0.05,0.32] and HEA-5: [0.35,0.15,0.05,0.11,0.34]. Error bars indicate the standard deviation of averaging over a set of SSOS solutions.

**Supplementary Table S2:** The top-five most stable compositions for AlCoCrFeNi HEA with an FCC lattice. The calculated formation energy, mass density and elastic moduli are compared with those of the BCC lattice at the same HEA composition. Error bars indicate the standard deviation of averaging over a set of SSOS solutions.

| **AlCoCrFeNi composition** | **Formation energy (eV/atom)** | | **Density (g/cm^3^)** | | **Lattice constant (Å)** | |
| --- | --- | --- | --- | --- | --- | --- |
|  | **FCC** | **BCC** | **FCC** | **BCC** | **FCC** | **BCC** |
| [0.35,0.17,0.05,0.08,0.35] | -0.264±0.003 | -0.280±0.005 | 6.57±0.01 | 6.61±0.01 | 3.627 ± 0.006 | 2.875±0.006 |
| [0.35,0.22,0.05,0.05,0.33] | -0.263±0.005 | -0.281±0.006 | 6.60±0.01 | 6.64±0.01 | 3.624 ± 0.005 | 2.871±0.005 |
| [0.35,0.17,0.05,0.09,0.34] | -0.261±0.004 | -0.277±0.006 | 6.55±0.01 | 6.60±0.01 | 3.629 ± 0.004 | 2.877 ± 0.003 |
| [0.35,0.23,0.05,0.05,0.32] | -0.261±0.004 | -0.279±0.005 | 6.61±0.01 | 6.64±0.01 | 3.623 ± 0.004 | 2.872±0.005 |
| [0.35,0.15,0.05,0.11,0.34] | -0.258±0.004 | -0.275±0.007 | 6.53±0.01 | 6.58±0.01 | 3.632 ± 0.004 | 2.877±0.005 |

**Supplementary Table S3:** The elastic moduli of the top-five most stable compositions of the AlCoCrFeNi HEA with an FCC lattice as compared with those of the AlCoCrFeNi HEA with a BCC lattice at the same composition. The Poisson coefficient for top five compositions of the AlCoCrFeNi HEA with an FCC lattice is ν=0.23. Error bars indicate the standard deviation of averaging over a set of SSOS solutions.

| **AlCoCrFeNi composition** | **Young’s modulus (GPa)** | | **Bulk modulus (GPa)** | | **Shear modulus (GPa)** | |
| --- | --- | --- | --- | --- | --- | --- |
|  | **FCC** | **BCC** | **FCC** | **BCC** | **FCC** | **BCC** |
| [0.35,0.17, 0.05,0.08,0.35] | 159±4 | 207±5 | 118±1 | 128±1 | 70±2 | 89±2 |
| [0.35,0.17,0.05,0.09,0.34] | 159±4 | 208±5 | 119±1 | 129±2 | 69±1 | 89±2 |
| [0.35,0.23,0.05,0.05, 0.32] | 161±4 | 215±4 | 112±2 | 122±1 | 74±2 | 93±2 |
| [0.35,0.17,0.05,0.11,0.32] | 157±3 | 207±5 | 117±1 | 129±2 | 689±2 | 89±2 |
| [0.35,0.17,0.05,0.14,0.29] | 156±3 | 207±4 | 116±1 | 127±1 | 69±1 | 89±2 |

# **Formation energy vs mass density**

The formation energies per atom vs the density for the AlCoCrFeNi HEA with BCC and FCC lattice are plotted in Figure S5 and Figure S6, respectively. Marker color indicates the molar fraction of Co (see Figure S5(a, b)), Cr (see Figure S5(c, d)), Fe (see Figure S6(a, b)) and Ni (see Figure S6(c, d)), while marker size indicates the molar fraction of Al. As can be seen in Figure S5 (a, b) the higher the molar fraction of Cr, the lower the density of the AlCoCrFeNi HEA (see the LD region in Figure S5). However, in contrast to Al, the lower the molar fraction of Cr, the lower the formation energy (see the LFE region in Figure 5 and Figure S5)).

There is a positive correlation between the formation energy and density as illustrated in Figure S5 and Figure S6. Nevertheless, the set of compositions with low formation energies (see the LFE region in Figure S5 and Figure S6)) and the set of low-density compositions (see the LD region in Figure S5 and Figure S6)) do not overlap. Therefore, an acceptable compromise between the low formation energy and the low density should be reached in the design of AlCoCrFeNi HEAs. Although for some constituent elements, such as Al, both the formation energy and the density of AlCoCrFeNi HEA can be lowered by increasing its molar fraction, for other constituent elements, such as Cr and Ni, one can either lower the density or formation energy by maximizing (or minimizing) molar fraction of the selected element. Therefore, the ultimate design of a AlCoCrFeNi HEA for specific applications requires finding a suitable compositional combination.


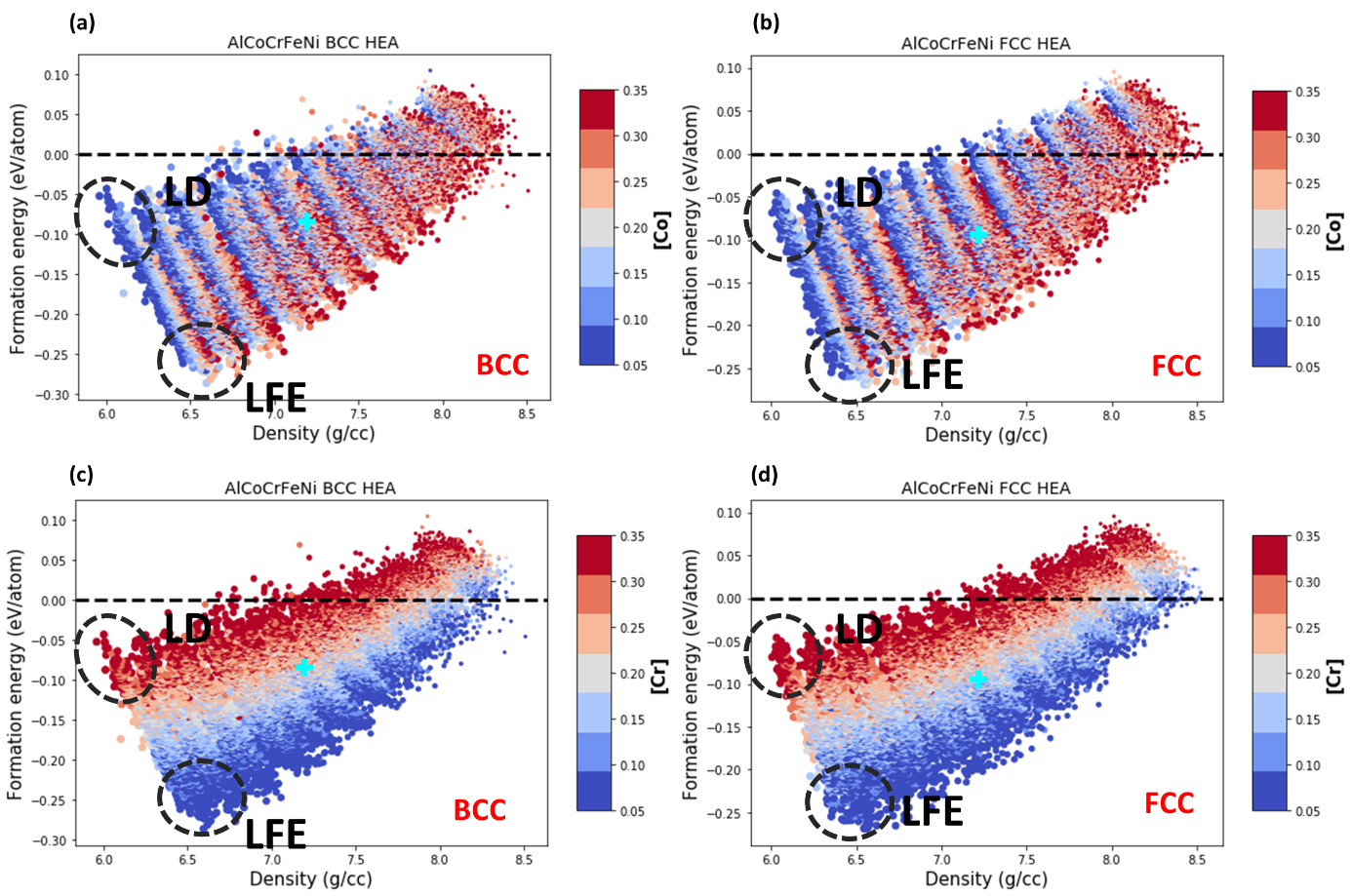


**Supplementary Figure S5**: The calculated formation energy per atom vs. the mass density for AlCoCrFeNi HEA with BCC and FCC lattice structure. Marker color indicates the molar fraction of Co in AlCoCrFeNi HEA with BCC (a) and FCC (b) lattice structure. Marker color indicates the molar fraction of Cr in AlCoCrFeNi HEA with BCC (c) and FCC (d) lattice structure. Marker size signifies the molar fraction of Al. Closed dashed line outlines the region of compositions with low densities (LD), and the region of compositions with low formation energies (LFE). The equimolar composition is indicated by cyan cross symbol.


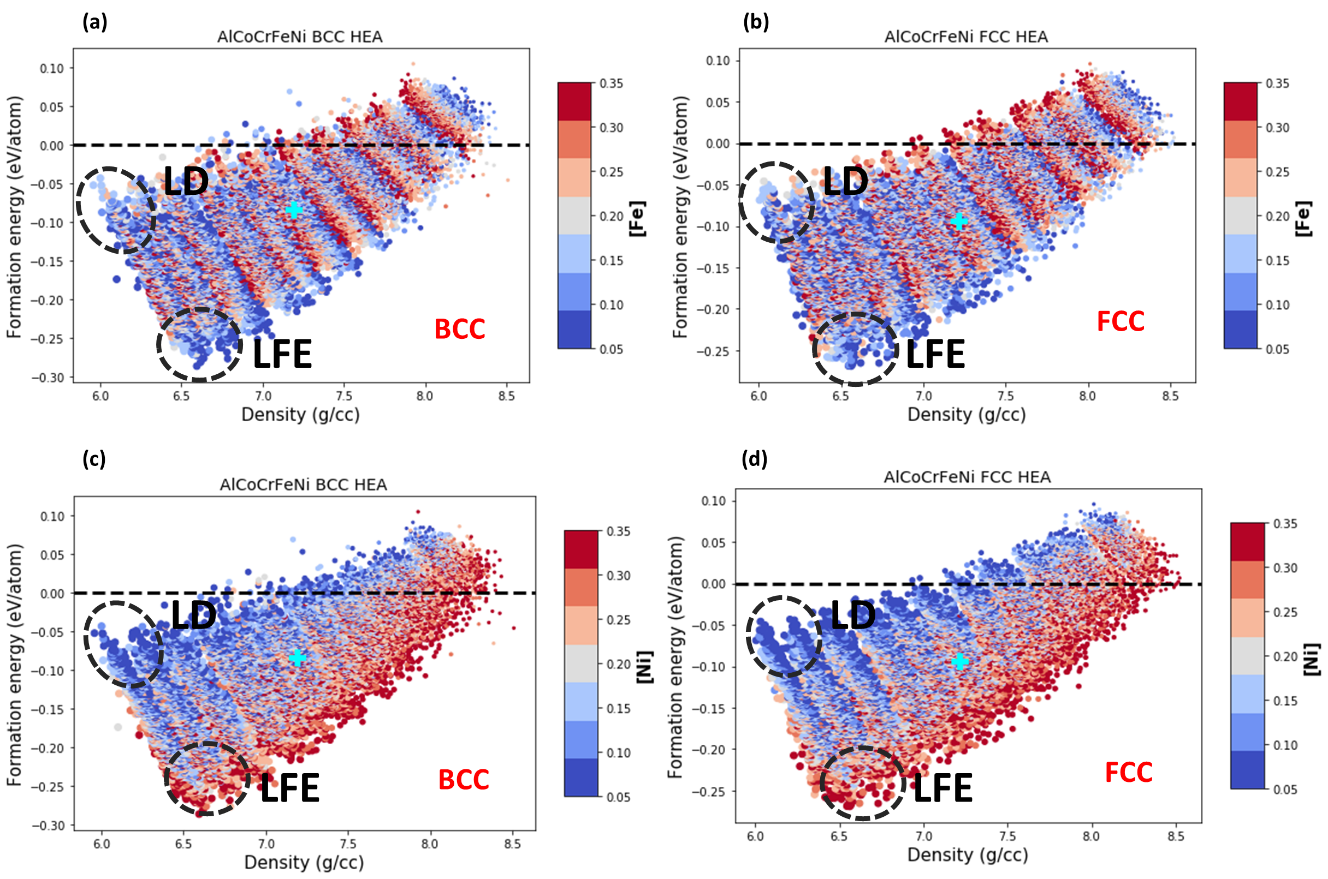


**Supplementary Figure S6:** The calculated formation energy per atom vs. the mass density for AlCoCrFeNi HEA with BCC and FCC lattice structure. Marker color indicates the molar fraction of Fe in AlCoCrFeNi HEA with BCC (a) and FCC (b) lattice structure. Marker color indicates the molar fraction of Ni in AlCoCrFeNi HEA with BCC (c) and FCC (d) lattice structure. Marker size shows the molar fraction of Al. Closed dashed line outlines the region of compositions with low densities (LD), and the region of compositions with low formation energies (LFE). The equimolar composition is indicated by cyan cross symbol.

# **Additional validation of the PSSOS method**

To check the validity of the PSSOS method, we compared stable solid solution phase predicted by the PSSOS for a given AlCoCrFeNi composition with the available experimentally measured results^1,2^ and those obtained by DFT-based coherent potential approximation (CPA)^3,4^. In addition, we also calculated the corresponding lattice constants for different compositions (see Figure S7).

In their experiments, Zhu et al.^1^ varied the molar fraction of Ni in a set of Ni_x_(AlCoCrFe)_1-x_ compositions. For each molar fraction of Ni, they determined the most stable phases (FCC, BCC, or both) and measured the corresponding lattice constant. First, we compared the phases predicted by the PSSOS method with the experimentally observed ones for Ni_x_(AlCoCrFe)_1-x_ compositions, and found an decent agreement. Second, we compared the values of lattice constant of the AlCoCrFeNi HEA calculated by the PSSOS method with the experimentally measured ones^1,2^ and by the CPA-based DFT method^3^ for both the FCC and BCC phases (see Figure S7). As can be seen in Figure S7, the values of lattice constants obtained by the PSSOS method for various compositions correspond well with the experimental ones and those obtained by DFT-based CPA approximation. We note that for Ni, in contrast to Al, the lattice constant decreases with an increase in its molar fraction. The overall agreements in the values of lattice constant calculated by the PSSOS method and obtained experimentally or by the CPA-based DFT add strong support to the present method.


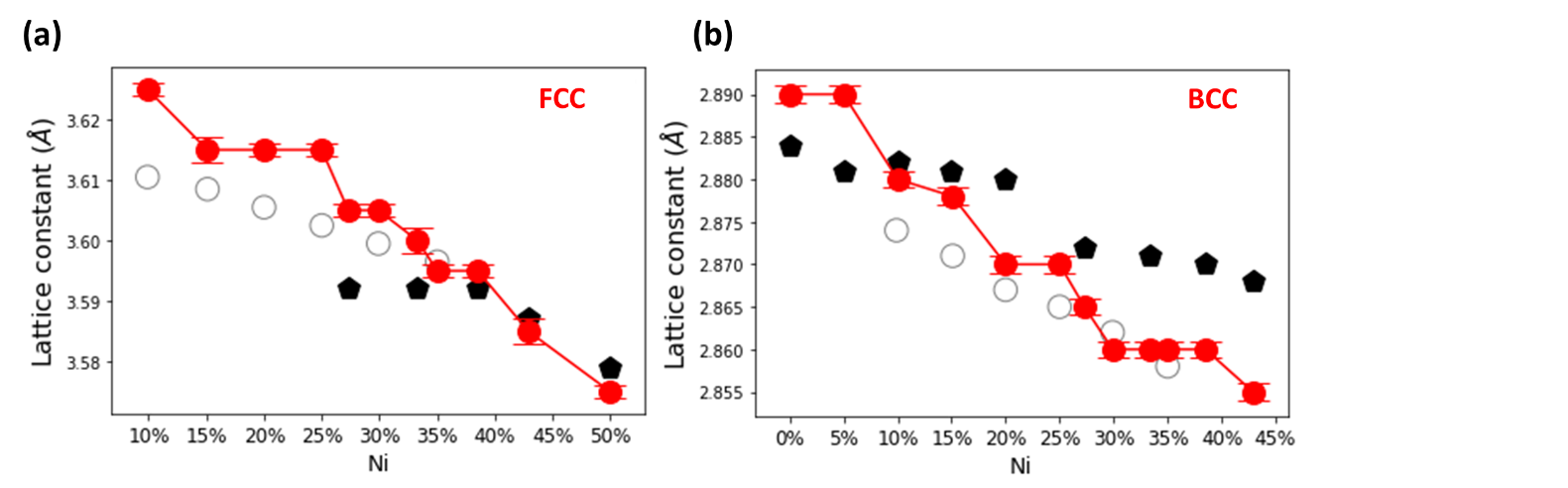


**Supplementary Figure S7:** The lattice constant of (AlCoCrFe)_1-x_Ni_x_ HEA in FCC (a) and BCC (b) phase vs. molar fraction of Al (%). The values of lattice constant obtained by the PSSOS method are represented by red circles, the error-bars indicate standard deviation, and the red line is to guide the eye. For comparison, the experimental measurements are taken from Zhu et al.^1^ (black pentagons) and the values obtained by the CPA-based DFT method are taken from Jasiewicz et al.^3^ (open circles).

Finally, we compared the values of lattice constant for a few specific non-equimolar compositions of the AlCoCrFeNi HEA with BCC and FCC lattice structure predicted by the PSSOS method and experimentally by Ma et al.^2^ (see Table S4). It is seen that again a good agreement is achieved.

**Supplementary Table S4:** Comparison of the lattice constants calculated by the PSSOS method and experimentally measured by Ma et al.^2^ for the selected non-equimolar compositions of the AlCoCrFeNi HEA with BCC and FCC lattices. Error bars indicate the standard deviation of averaging over a set of SSOS solutions. The relative error is indicated in the last column.

| **Composition [AlCoCrFeNi]** | **Lattice structure** | **Experiment: Lattice constant (Å)** | **PSSOS: Lattice constant (Å)** | **Error (%)** |
| --- | --- | --- | --- | --- |
| [0.125,0.25,0.1875,0.1875,0.25] | BCC | 2.874 | 2.86±0.002 | 1% |
| [0.125,0.175,0.175,0.35,0.175] | BCC | 2.862 | 2.85±0.001 | 1% |
| [0.125,0.25,0.1875,0.1875,0.25] | FCC | 3.593÷3.603 | 3.56±0.003 | 1% |
| [0.125,0.175,0.175,0.35,0.175] | FCC | 3.572÷3.584 | 3.58±0.002 | -1% |

# Pair correlation functions


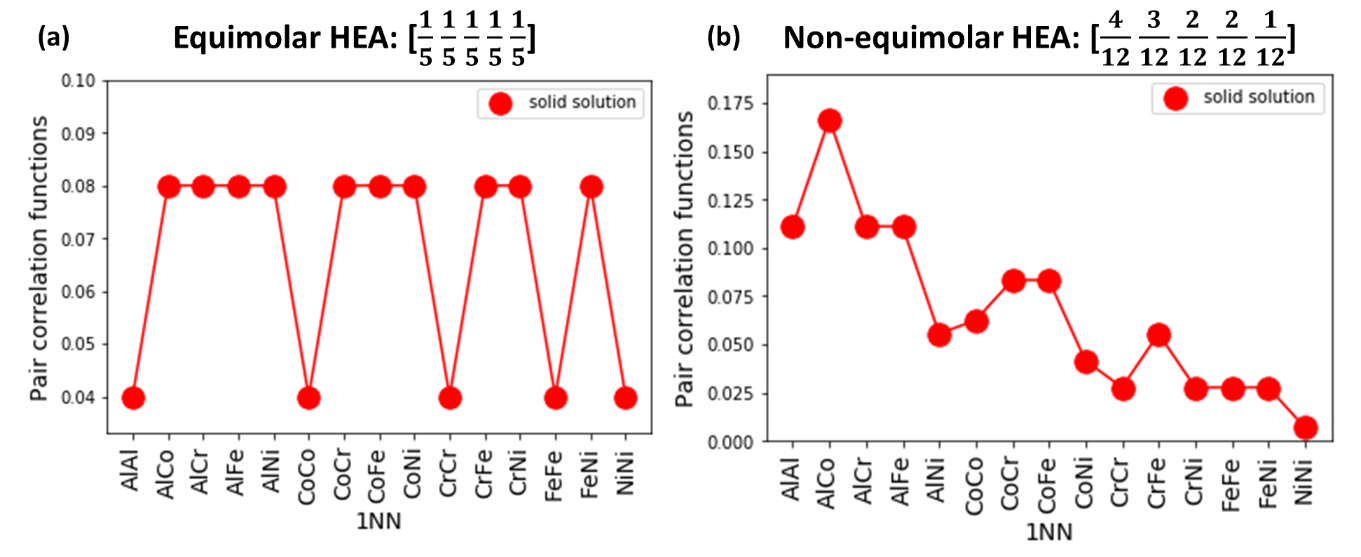


***Supplementary Figure S8:*** *The pair correlation functions within the 1^st^ NN for AlCoCrFeNi HEA equimolar sample(a) with composition* *[c(Al),c(Co),c(Cr),c(Fe),c(Ni)] = [*$\frac{1}{5},\frac{1}{5},\frac{1}{5},\frac{1}{5},\frac{1}{5}$*] and non-equimolar sample (b) with composition [c(Al),c(Co),c(Cr),c(Fe),c(Ni)] = [*$\frac{4}{12},\frac{3}{12},\frac{2}{12},\frac{2}{12},\frac{1}{12}$*].*

# **References**

1. Zhu, Z. G., Ma, K. H., Wang, Q. & Shek, C. H. Intermetallics Compositional dependence of phase formation and mechanical properties in three CoCrFeNi- ( Mn / Al / Cu ) high entropy alloys. **79**, 1–11 (2016).

2. Ma, Y. *et al.* Chemical short-range orders and the induced structural transition in high-entropy alloys. *Scr. Mater.* **144**, 64–68 (2018).

3. Jasiewicz, K., Kaprzyk, S. & Tobola, J. Interplay of Crystal Structure Preference and Magnetic Ordering in High Entropy CrCoFeNiAl Alloys. *ACTA Phys. Pol. A* **133**, 511–513 (2018).

4. Tian, F. *et al.* Structural stability of NiCoFeCrAlx high-entropy alloy from ab initio theory. *Phys. Rev. B* **88**, 085128 (2013).
